# Teleportation of a genuine single-rail vacuum-one-photon qubit generated via a quantum dot source

**DOI:** 10.1038/s44310-024-00046-1

**Published:** 2024-11-29

**Authors:** Beatrice Polacchi, Francesco Hoch, Giovanni Rodari, Stefano Savo, Gonzalo Carvacho, Nicolò Spagnolo, Taira Giordani, Fabio Sciarrino

**Affiliations:** Dipartimento di Fisica, Sapienza Università di Roma, Roma, Italy

**Keywords:** Quantum physics, Quantum information, Quantum mechanics, Qubits, Single photons and quantum effects, Quantum optics, Single photons and quantum effects

## Abstract

Quantum state teleportation represents a pillar of quantum information and a milestone on the roadmap towards quantum networks with a large number of nodes. Successful photonic demonstrations of this protocol have been carried out employing different qubit encodings. However, demonstrations in the Fock basis encoding are challenging, due to the impossibility of generating a coherent superposition of vacuum-one photon states on a single mode with linear optics. Indeed, previous realizations only allowed the teleportation of dual-rail entangled states, by exploiting ancillary electromagnetic modes. Here, instead, we enable the quantum teleportation of pure vacuum-one-photon qubits encoded in a single spatial mode, by exploiting coherent control of a resonantly excited semiconductor quantum dot in a micro-cavity. Within our setup, we can both teleport genuine single-rail vacuum-one-photon qubits and perform entanglement swapping. Our results may disclose new quantum information processing potentialities for this encoding, whose manipulation is achievable via quantum dot single-photon sources.

## Introduction

Quantum teleportation^1–3^ is one of the most intriguing processes arising from the theory of quantum mechanics, being at the core of quantum technologies as well as to the development of many concepts in quantum information theory. This protocol is enabled by a shared quantum entangled resource. A quantum state is jointly measured with one-half of the entangled resource in a given place and, as a result of such an operation, it is transferred to the other half in a remote location. Such a protocol, together with entanglement swapping^4,5^, is at the core of several quantum computation^6,7^ and quantum communication schemes ranging from quantum repeaters^8,9^, quantum gate teleportation^10–12^, measurement-based quantum computing^13–15^ as well as port-based quantum teleportation^16,17^. Over the years, several experiments have successfully shown the teleportation of unknown quantum states using different experimental setups and degrees of freedom^2,3,18–25^. In particular, the first platforms were based on photonic systems^2,3^, since photons represent natural information carriers, due to their low interaction with the environment and to the capability of their efficient manipulation through linear optics. Quantum teleportation has been implemented over hundreds of kilometers using free-space channels^26,27^, across metropolitan networks^28–30^, using satellites stations^31^ and near-deterministic single-photon sources^32^. In this context, a major challenge consists in teleporting qubits encoded in the photon-number basis. We identify two main reasons. Firstly, this encoding is affected by photon losses. Secondly, generating photon-number superposition states on a single electromagnetic mode is unfeasible only by means of linear optical elements. Nowadays, these two major experimental challenges can be addressed by near-deterministic single-photon sources that proved to reach high level of efficiency^33,34^ and to generate genuine single-rail vacuum-one-photon states^35,36^. Furthermore, the recent progress in the development of high-efficiency single-photon detectors^37^ and low-loss optical platforms^38^ open new perspectives for the investigation of information encoding based on the number of photons.

Previous quantum teleportation implementations in the photon-number basis^21,25^ considered the teleportation of a vacuum-one-photon qubit produced by making a photon impinging on a beam splitter and encoding the qubit into one of the two output modes. In detail, the output state is entangled over the two output modes of the beam splitter. One mode is used to encode the qubit to be teleported while the other mode encodes an ancillary qubit used for the final verification of the protocol. Consequently, as also stated by the authors, the teleported state is, in fact, not a *genuine* vacuum-one-photon qubit, but a subsystem of an entangled state, making this scheme corresponding precisely to entanglement swapping.

Here, we propose a way to overcome such limitations, based on the nonlinear features enabled by the resonant excitation of a quantum dot single-photon source^39,40^. In detail, the main novelty of our work lies in the first quantum teleportation of arbitrary pure vacuum–one-photon qubits genuinely encoded on a single electromagnetic mode. Recent advances in quantum dot (QD) sources technology have enabled a significant step forward in the generation of photonic states for quantum information protocols^41–47^. Our approach relies on the generation of single photons through resonant optical excitation of a QD in a microcavity^33,42^, which was recently shown to be able to produce coherent superposition states of vacuum and one photon^35^, as well as photon-number entangled states^36^. This is due to its capability of transferring the coherence of the excited-ground atomic state to the generated photons^35,42^. By harnessing this property, we were able to precisely control the vacuum-one-photon state to be teleported, as shown by the achieved results.

Indeed, we first design a scheme tailored to the teleportation of genuine single-rail vacuum–one-photon qubits. Then, we compare our teleportation scheme with the entanglement swapping one, by implementing also the latter in the spirit of the previous demonstrations of teleportation using the vacuum-one-photon paradigm^21,25^. These results demonstrate a step forward with respect to previous works where only entangled states could be teleported. The results are highly compatible with the expectations, demonstrating that our platform allows for the teleportation of both entangled and single-qubit states in the Fock basis encoding.

Our experiment represents a step forward in the field of quantum communication, being the first example of genuine quantum teleportation of a pure state in the Fock basis, and stimulating further investigations in protocols involving photon-number encoded states^36^ for quantum information tasks. Furthermore, our results may offer exciting prospects for the development of QD-based advanced quantum technologies.

## Results

### Quantum state teleportation

This section is divided into four paragraphs. In the first one, we recall the standard circuital description of the quantum teleportation protocol. In the second one, we translate the circuital paradigm into our photonic protocol proposal where logical qubits are encoded in the Fock basis. In the third paragraph, we describe our experimental platform for the implementation of the proposed protocol. Finally, in the fourth paragraph, we discuss our experimental results.

#### Theoretical Background

**Fig. 1:**
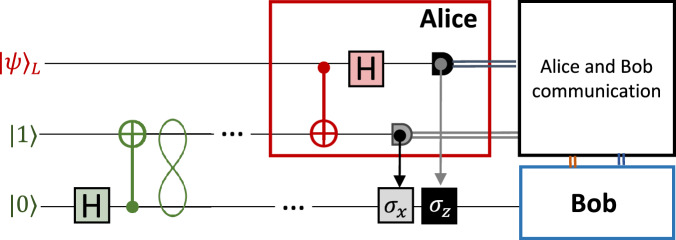
Circuit of the quantum teleportation protocol. The Bell state generated by the Hadamard (H) and CNOT gates (in green) is shared between Alice and Bob. Alice performs a Bell measurement (in red) between the qubit $\left\vert \psi \right\rangle$ to teleport and one of the two qubits in the Bell state. Bob retrieves the qubit $\left\vert \psi \right\rangle$ by applying *σ*_*x*_ and/or *σ*_*z*_ according to Alice’s measurement outcomes.

Before going into the details of the experiment, we briefly review the quantum teleportation protocol through its circuital representation, shown in Fig. 1.

In the quantum teleportation protocol, Alice aims at sending a generic qubit of the form:

1 $$\left\vert \psi \right\rangle =\alpha\, {\left\vert 0\right\rangle }_{L}+\beta\, {\left\vert 1\right\rangle }_{L},$$

to Bob, in absence of a direct quantum channel, by only exploiting a classical channel and a shared Bell state. Here, the index *L* is used to indicate the logical qubit. From a circuital point of view, reported in Fig. 1, the Bell state is produced by applying the following transformation to an initial two-qubit state:

2 $$\begin{array}{ll}\left\vert {\psi }^{+}\right\rangle \,=\,\,\text{CNOT}(\text{H}\otimes \text{I}\,)\,{\left\vert 01\right\rangle }_{L}\\ \quad\quad\;\;=\,\displaystyle\frac{{\left\vert 01\right\rangle }_{L}+{\left\vert 10\right\rangle }_{L}}{\sqrt{2}}\end{array}$$

To teleport her state, Alice performs a Bell-state measurement (BSM) between the two qubits on her stage, i.e., $\left\vert \psi \right\rangle$ and one half of the Bell state, obtaining a two-bit outcome. Depending on the result of such an operation, the second half of the Bell state, sent to Bob’s stage, is left in one of the possible states: $\alpha {\left\vert 0\right\rangle }_{L}+\beta {\left\vert 1\right\rangle }_{L},\alpha {\left\vert 1\right\rangle }_{L}+\beta {\left\vert 0\right\rangle }_{L},\alpha {\left\vert 0\right\rangle }_{L}-\beta {\left\vert 1\right\rangle }_{L}$, or $\alpha {\left\vert 1\right\rangle }_{L}-\beta {\left\vert 0\right\rangle }_{L}$. Therefore, to retrieve the original state $\left\vert \psi \right\rangle$, Bob may need to apply a unitary transformation to his state according to Alice’s two-bit outcome.

#### Quantum state teleportation in the vacuum-one-photon encoding

**Fig. 2:**
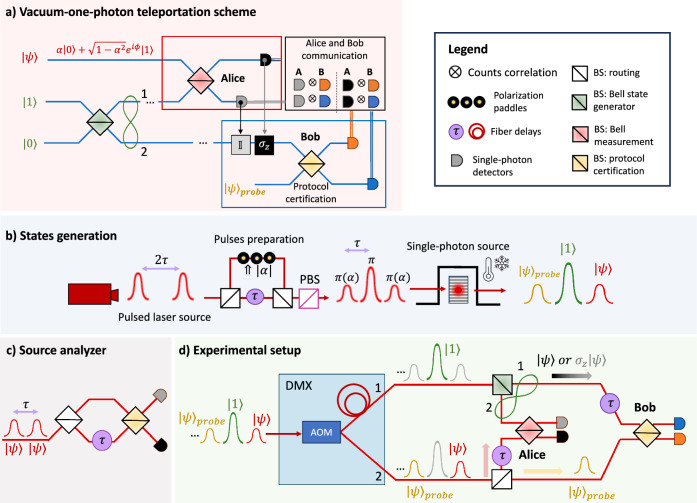
Quantum teleportation protocol in the vacuum–one-photon encoding. **a** Circuit realization of a probabilistic quantum teleportation protocol in the vacuum-one-photon qubit encoding. The Bell state generation (green) and a probabilistic Bell measurement (red) are realized through the interference of single photons in a beam-splitter (BS). Alice communicates the outcomes of the measurement to Bob. Bob applies accordingly the identity or *σ*_*z*_ and certifies the teleportation by a self-homodyne detection with a reference state performed by the last yellow BS. **b** In the first step of our experimental setup, we double the repetition rate of the pump pulsed laser, from ~80 Mhz to ~160 Mhz, through an in-fiber Mach-Zehnder interferometer (MZI). Polarization paddles control the pump power of the pulses after passing through a polarizing beam-splitter (PBS). Such a modulation of the pulses power allows to resonantly excite our QD to generate sequence of states like ${\left\vert \psi \right\rangle }^{t = 0},{\left\vert 1\right\rangle }^{t = \tau }$ and ${\left\vert \psi \right\rangle }_{probe}^{t = 2\tau }$, where $\left\vert \psi \right\rangle$ is the arbitrary qubit of Eq. (3). **c** The MZI employed to characterize the vacuum-one-photon qubits generated by the source, i.e., the estimate of the *α* value from the visibility of the interference fringes. **d** The train of states is distributed and synchronized in two different channels by a time-to-space de-multiplexer (DMX) based on an acoustic-optical modulation (AOM). In the first channel (*ch*_1_), the ${\left\vert 1\right\rangle }^{t = \tau }$ state is sent through the green BS to generate a Bell state, and, afterward, half of it interferes at time *t* = *τ* in Alice’s station with $\left\vert \psi \right\rangle$ coming from the second channel (*ch*_2_). The second half of the Bell state, i.e., the teleported state, is sent to Bob, who characterizes it through interference at time *t* = 2*τ* with ${\left\vert \psi \right\rangle }_{{probe}}$ that is a copy of the original qubit $\left\vert \psi \right\rangle$. The red, yellow, black arrows represent the case in which the protocol succeeds due to the probabilistic routing of $\left\vert \psi \right\rangle$ and ${\left\vert \psi \right\rangle }_{{probe}}$, as all pulses take the correct delay lines.

In Fig. 2a, we show the conceptual scheme of our quantum teleportation experiment, that implements the teleportation of a vacuum-one photon state, i.e., where the logical qubits ${\left\vert 0\right\rangle }_{L}$ and ${\left\vert 1\right\rangle }_{L}$ are encoded in the physical Fock states with zero and one photon, respectively, i.e., ${\left\vert 0\right\rangle }_{L}:=\left\vert 0\right\rangle$ and ${\left\vert 1\right\rangle }_{L}:=\left\vert 1\right\rangle$. In detail, we initially generate a genuine vacuum-one photon qubit in the state:

3 $$\left\vert \psi \right\rangle =\alpha\, \left\vert 0\right\rangle +\sqrt{1-{\alpha }^{2}}{e}^{i\phi }\left\vert 1\right\rangle$$

that is equivalent to the one in Eq. (1). We suppose that the coefficient *α* is a real number. Moreover, we take the phase *ϕ* as a reference and, therefore, for simplicity, we can assume that it amounts to zero. The one-photon Fock state $\left\vert 1\right\rangle$ is used to produce the Bell state shared between Alice and Bob as required by the teleportation protocol, by sending it onto a symmetric BS with modes labeled as 1 and 2. This optical element transforms the creation operators according to ${a}_{1}^{\dagger }\longrightarrow ({a}_{1}^{\dagger }+{a}_{2}^{\dagger })/\sqrt{2}$, so that the resulting state reads:

4 $$\left\vert {1}_{1}{0}_{2}\right\rangle \longrightarrow \frac{\left\vert {1}_{1}{0}_{2}\right\rangle +\left\vert {0}_{1}{1}_{2}\right\rangle }{\sqrt{2}}$$

In the vacuum-one photon encoding previously defined, the state above is a maximally entangled state equivalent to the one in Eq. (2). The subsystem in mode 1 is sent to Alice while the subsystem in mode 2 is sent to Bob. In her station, Alice performs a partial BSM between the qubit $\left\vert \psi \right\rangle$ and her half of the Bell state by means of the red symmetric BS. After Alice’s BSM, the half of the Bell state held by Bob in mode 2 is left with a success probability *p*_±_ = 1/4 either in the state $\left\vert \psi \right\rangle$ or ${\sigma }_{z}\left\vert \psi \right\rangle$, depending on Alice’s measurement outcome. Finally, Bob validates the protocol by performing a suitable measurement between the received qubit and a reference state ${\left\vert \psi \right\rangle }_{{probe}}$, which is only used as a probe to certify the protocol success. This measurement aims at witnessing the coherence between the two logical states, i.e., the vacuum and the one-photon components. As ${\left\vert \psi \right\rangle }_{{probe}}$, we employ a copy of the original qubit $\left\vert \psi \right\rangle$ of Eq. (3). Such a method, known as self-homodyne detection, allows measuring coherence by observing interference fringes in the single-photon counts at the output of the BS, as a function of the relative phase between the optical paths. The presence of such interference fringes is a signature of the coherence in the input states, since it cannot be obtained by mixed states. Additionally, its observation requires the preparation of indistinguishable photons to enable quantum interference. Furthermore, the visibility of such fringes depends on the vacuum population of the two qubits^35^. Such a method can be used to characterize the output of the teleportation protocol, thus verifying that it has been carried out successfully. Further descriptions of the self-homodyne detection in our protocol can be found in Supplementary Notes I–IV.

#### Experimental apparatus

Our experimental setup is depicted in Fig. 2b–d. As a single-photon source, we use a commercially available (Quandela *e-Delight*) semiconductor QD. Such source consists of an InGaAs matrix placed in a nanoscale electrically controlled micropillar cavity^42^ kept at cryogenic temperature (around 4K) by an *Attocube-Attodry800* He-closed cycle cryostat. The QD is optically excited by a pulsed laser resonant with the cavity characteristic wavelength (928.05 nm), in the resonance fluorescence (RF) regime^33,42^. We achieve single-photon collection by means of a single-mode fiber (SMF) located inside the cryostat (collection efficiency ≈ 10%) and detect photons with avalanche photodiodes (APDs) with efficiency amounting to ≈30%. Photons are separated from the residual pumping laser in a cross-polarization scheme. The nominal repetition rate of the laser amounts to 79 MHz, but we double it by introducing a delay between two consecutive pulses and then recombining them, as shown in the “Pulses preparation” section of Fig. 2b. The procedure of doubling the laser repetition rate is key to our protocol. Indeed, such a preparation stage allows us to control the polarization of either pulse independently and, consequently, their power, as the laser light passes through a polarizing beam splitter (PBS) before optically exciting the QD. This is needed because, in the RF excitation scheme, an excitation power lower than the *π*-pulse translates into the generation of coherent vacuum-one photon states of the same form of Eq. (3), where the population of the state $\left\vert 1\right\rangle$, i.e., 1 − *α*^2^, grows with the excitation power^35^, achieving the value 1 at the *π*-pulse. Therefore, as depicted in Fig. 2b, by independently manipulating the power of the two pulse trains, we make the source generate two different kinds of states, namely the qubit to be teleported $\left\vert \psi \right\rangle$, which is arbitrarily chosen, its probe copy ${\left\vert \psi \right\rangle }_{probe}$, and the one-photon Fock state $\left\vert 1\right\rangle$, used to share entanglement between Alice and Bob, as explained in the previous section.

In the Methods section, we report the typical performances of the source measured with the Mach-Zehnder interferometer (MZI) of Fig. 2c for what concerns the count rate, the second-order auto-correlation function *g*^(2)^, the HOM visibility and the conditional purity of the generated vacuum-one photon qubits, which is the purity conditioned on the success of the protocol (for further information see “Methods” and Supplementary Note I).

In our protocol, we have two characterization stages. Before the teleportation protocol, we characterize the qubit $\left\vert \psi \right\rangle$. Then, after the teleportation protocol, Bob characterizes the teleported qubit by means of a probe copy of qubit $\left\vert \psi \right\rangle$, denoted as ${\left\vert \psi \right\rangle }_{probe}$. The details of such characterizations are explained in the following.

Characterization of the qubit $\left\vert \psi \right\rangle$ is performed independently by using a time-unbalanced MZI, as the one shown in Fig. 2c. More specifically, a train of states prepared in the target state, characterized by amplitude *α* for the vacuum component, is prepared in the same spatial mode and injected in one input port of a symmetric BS. A delay *τ*, equal to the time separation between two input pulses, is then applied in one of the output modes of the BS. Then, the output modes interfere in a second symmetric BS, and the output fringes are measured from the single-photon counts in each of the output modes, as a function of the relative free evolving phase in the interferometer. The visibility *V* of such a pattern provides a direct estimate of *α*^2^ (see “Methods” and Supplementary Note I).

For the teleportation protocol, we set the two laser pulse trains at two different powers to produce alternatively the states $\left\vert \psi \right\rangle$ and $\left\vert 1\right\rangle$, respectively at times *t* = *nτ* and *t* = (*n* + 1)*τ*, where *n* = 0, 1, … and *τ* ≈ 6 ns. We trigger each protocol run in a way such to take as timing reference the following sequence: ${\left\vert \psi \right\rangle }_{{probe}}^{t = 2\tau },{\left\vert 1\right\rangle }^{t = \tau }$, and ${\left\vert \psi \right\rangle }^{t = 0}$. The generated states are sent through a Quandela commercially available temporal-to-spatial demultiplexer (DMX) which actively separates the incoming pulses into two synchronized but spatially different ≈150 ns-long trains (see Fig. 2d). The ${\left\vert 1\right\rangle }^{t = \tau }$ state brought by the DMX channel 1 is sent on a BS to produce the Bell state in Eq. (4). Details about the generation of the experimental Bell state and its characterization are discussed in Supplementary Note II. One half of such Bell state is sent to Alice, who performs a partial BSM between it and the ${\left\vert \psi \right\rangle }^{t = 0}$ state brought by the DMX channel 2. The second half of the Bell state is sent to Bob, who characterizes it through interference with the ${\left\vert \psi \right\rangle }_{{probe}}^{t = 2\tau }$ state, also brought by the DMX channel 2. More specifically, we record Bob’s single counts traces conditioned on the presence of one photon in Alice’s station ≈6 ns before, by taking a two-fold coincidence window equal to 1.5 ns. Note that the routing of $\left\vert \psi \right\rangle$ and ${\left\vert \psi \right\rangle }_{{probe}}$ is not deterministic, thus lowering the success probability to *p*_±_ = 1/16.

#### Teleportation results

Here, we report and discuss our experimental results for the quantum teleportation protocol.

**Fig. 3:**
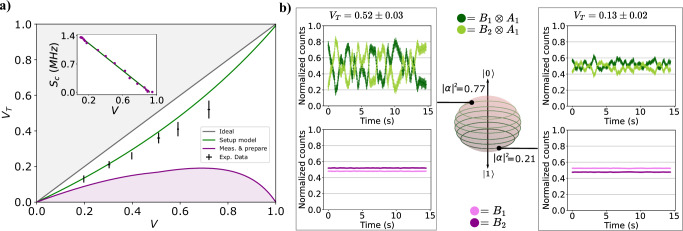
Theoretical expectations and experimental results for the teleportation protocol. **a** In gray, we show the expected visibility *V*_*T*_ of the teleported state as a function of the probe state visibility *V* within an ideal platform, with deterministic routing in every MZI, photon-number resolving detectors, the same self-homodyne measurement station, and perfect photon indistinguishability. In green, we show how such expectations change when considering that, in our platform, the separation of the probe and the qubit to be teleported is not deterministic, and threshold detectors are employed. Furthermore, this model is formulated in the limit of high losses and considering imperfections such as the state conditional purity and partial photon distinguishability. In purple, instead, we report the same quantities when using the fully classical *measure-and-prepare* protocol, which allows for the highest fidelity achievable with only classical resources^51^ in the same lossy regime. In the inset, we report the single count rate (*S*_*c*_) in one of two outputs of the self-homodyne station (see Fig. 2c) as a function of the visibility, for power pulse areas below or equal to *π*. From this plot, we estimate a state conditional purity *λ* ≈ 0.98. **b** Two examples of the free evolution of the single-photon counts observed on Bob’s detectors after the teleportation protocol and corresponding to two different states. The green plots show events in Bob’s station, conditioned on the presence of one photon in Alice’s detector number 1. In pink, instead, we show the unfiltered single count traces detected on Bob’s side. Each pair of traces is normalized to the sum of the two single-photon signals. In this figure, we also illustrate that the states we are able to teleport correspond to parallel planes on the Bloch sphere, since no phase information can be retrieved with our measurements. All the shown uncertainties amount to one standard deviation and were computed by assuming Poissonian events.

To demonstrate that the teleportation protocol was successful, we analyzed the interference fringes which are obtained by the self-homodyning technique^35^ described above. More specifically, the main concept is to have a quantum state, in principle unknown and that needs to be characterized, interfere on a beam splitter with a reference probe prepared in a precise state such as the qubit in Eq. (1). It can be shown (as discussed in the Methods and in Supplementary Note I) that the visibility of interference fringes leads to a direct estimation of the vacuum population and the conditional purity of the unknown state. Moreover, the presence of interference fringes is a genuine signature of the coherence between the vacuum $\left\vert 0\right\rangle$ and single-photon term $\left\vert 1\right\rangle$ in the state, given that an input mixed state results in no interference in the output pattern as a function of the phase between the modes. The standard figure of merit to assess the quality of a quantum teleportation protocol is the average fidelity between teleported and target states. However, as investigated in refs. ^48,49^, the average fidelity can be lower than the classical bound even if genuine quantum resources were used to implement teleportation and alternative witnesses of nonclassicality can be found^50^. Therefore, to demonstrate the nonclassicality of our protocol, we opted for comparing the fringe visibility of the teleported state *V*_*T*_ with the target’s one *V*. Such a choice is motivated by observing that the relation between *V*_*T*_ and *V* has different behaviors in the quantum and in the classical case. In particular, as we demonstrate in Supplementary Note III, the visibilities that would be achieved with a fully classical *measure-and-prepare* strategy, which allows for the highest fidelity achievable with only classical resources^51^, define a bounded region, highlighted in purple in Fig. 3a, which has no intersection with the visibility achievable by using quantum resources. Moreover, our experimental points are compatible with the theoretical expectations within at least two standard deviation in the worst case. In contrast, the distance from the *measure-and-prepare* model is always larger than two standard deviation even in the region where the quantum and the classical curves are expected to be close. In summary, the teleported visibility achievable with quantum resources violates the classical limits defined by the region accessible through the *measure-and-prepare* strategy. Therefore, it represents a quantitative figure of merit that can be used to demonstrate genuine quantum teleportation as soon as the experimental visibilities lie outside such a region.

Our results are shown in Fig. 3. In the inset of Fig. 3a, we report the data used to extrapolate the conditional purity of the generated states, which amounts to ≈ 0.98, as explained in the Methods section. In detail, we show the relationship between *V* and the single-count rate recorded by one of the two detectors at the output of the last BS, when varying the vacuum population *α*^2^, i.e the power of the pump pulses. In the case of lossy apparatuses and identical pulses, such to generate trains of qubits with the same *α*, it can be shown that both the *V* and single-photon counts are linearly proportional to *α*^2^ (see Methods and Supplementary Note I for the full derivation). In the main plot of Fig. 3a, instead, we report the experimental results of the teleportation protocol, represented as black dots. We compare our observations with three different theoretical models for the teleported visibility *V*_*T*_ of the fringes in Bob’s counts conditioned by Alice’s measurements as a function of the probe state visibility *V*. In the first two models (gray and green curves) we assume correct teleportation, i.e the teleported state is an exact copy of $\left\vert \psi \right\rangle$. In gray, we show the trend *V*_*T*_ = *V* that would be observed if our setup was equipped with fully deterministic routing of $\left\vert \psi \right\rangle$ and ${\left\vert \psi \right\rangle }_{{probe}}$, as in the scheme in Fig. 2a, and photon-number resolving detectors. In the green plot, instead, we show a model taking into account the features of our platform, where deterministic routing is only applied to separate the central pulse from the two copies of the $\left\vert \psi \right\rangle$ state, and threshold detectors are employed. Furthermore, we also consider the imperfections of the source for what concerns the qubits conditional purity and the partial photon distinguishability (see Methods and Supplementary Note IV for the full derivation). Finally, the purple region corresponds to what would be observed in the case of the *measure-and-prepare* protocol^51^, as we demonstrate in Supplementary Note III. In this case, the classically teleported (CT) state would be the following statistical mixture: ${\rho }_{CT}=\frac{1}{3}\left\vert \psi \right\rangle \left\langle \psi \right\vert +\frac{1}{3}\left\vert 0\right\rangle \left\langle 0\right\vert +\frac{1}{3}\left\vert 1\right\rangle \left\langle 1\right\vert$ that corresponds to the closest-to-target state that can be achieved classically, having a fidelity *F* = 2/3. Our experimental observations are compatible with the green model within two standard deviations and violate the *measure-and-prepare* bound thus showing that our protocol outperforms its best classical counterpart.

Moreover, to demonstrate another pivotal element of quantum teleportation, i.e., the necessity of classical communication between Alice and Bob, in Fig. 3b, we report in green (violet) our protocol performances when (not) allowing classical communication between Alice and Bob, for two different teleported coefficients *α*^2^. In the green plots, where Bob’s single-photon counts are conditioned on the presence of one photon on one of Alice’s detectors, we observe free-evolving fringes corresponding to successful teleportation. In the violet plots, instead, where Bob’s single-photon counts are unfiltered, we cannot observe any fringes, due to lack of information about Alice’s measurement outcome. The same plot illustrates that we teleport quantum states lying on a plane of the Bloch sphere, as no phase information can be retrieved with our measurements.

In the next section, we show the implementation of an entanglement swapping protocol within the apparatus. The results we are going to present have a two-fold goal. On one hand, they highlight the difference between the platform needed for teleportation and the one needed for entanglement swapping in the vacuum–one-photon encoding, which share the same quantum channel. On the other hand, they allow us to obtain a further certification of the teleportation protocol since, from the swapping results, we retrieve additional information about the quality of the quantum channel that was employed to implement both the genuine teleportation and the swapping protocols.

### Entanglement swapping in the vacuum–one-photon encoding

This section is divided into three paragraphs. In the first one, we recall the circuital description of the entanglement swapping protocol and its photonic implementation. In the second one, we describe our experimental setup for the implementation of the protocol. In the third section, we discuss our experimental results.

#### Theoretical background

**Fig. 4:**
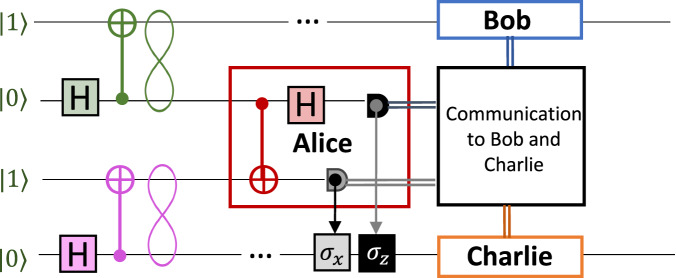
Circuital scheme of the entanglement swapping protocol. Alice performs a Bell measurement on two qubits that belong to two independent Bell states. Bob and Charlie will share a Bell state after the communication of Alice’s measurement outcomes and the consequent applications of *σ*_*x*_ or/and *σ*_*z*_ operators on Charlie’s qubit.

In the entanglement swapping protocol, whose scheme is shown in Fig. 4, entanglement is transferred from a given pair of particles to another at distant nodes. In detail, two independent Bell pairs are initially generated. One-half of both pairs is sent to a central observer, Alice, who performs a BSM. The remaining particles, each belonging to initially distinct pairs, are sent to two arbitrarily distant parties, Bob and Charlie. The interaction of the two subsystems in Alice’s station results in the state shared by Bob and Charlie ending up in a maximally entangled state. As in the quantum teleportation protocol, Bob and Charlie may need to apply a unitary transformation according to Alice’s outcome to retrieve the initial Bell state.

**Fig. 5:**
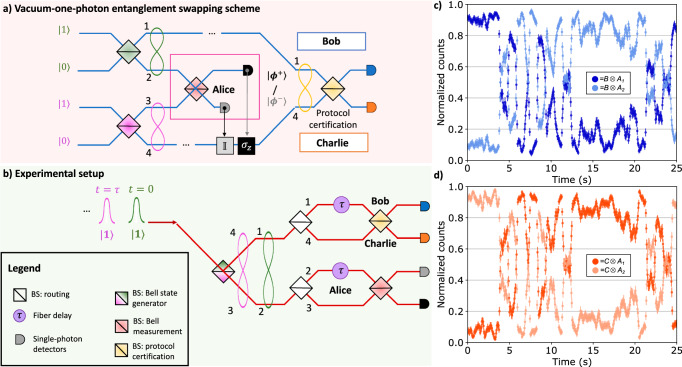
Entanglement swapping in the vacuum-one-photon encoding. **a** Entanglement swapping in the vacuum-one-photon encoding. Two single photons enter in two different BSs (in green) that generate two Bell states. Alice performs the probabilistic Bell measurement (red BS) and communicates to Charlie and Bob which detector clicks. Then, Charlie applies the identity or *σ*_*z*_. Bob and Charlie perform a further probabilistic Bell measurement to identify the entangled state they share. **b** We prepared a train of $\left\vert 1\right\rangle$ states and generated Bell states at times *t* = 0 and *t* = *τ* on the first BS. Alice performs a BSM by making interfere half of the Bell state generated at *t* = 0 with half of the next entangled state generated at *t* = *τ*. Bob and Charlie perform the same measurement to verify the success of the entanglement swapping protocol after the communication of Alice’s station outcomes. **c** Freely evolved fringes recorded by Bob’s detector (blue and light blue data) and (**d**) by Charlie’s one (red and light red traces), triggered by the two Alice’s BSM outcomes. Each pair of traces is normalized to the sum of the two single-photon signals. The uncertainties derive from the Poissonian statistics of single-photon counts.

In what follows, we present and demonstrate a probabilistic version of the entanglement swapping protocol encoded in the photon-number basis, which is depicted in Fig. 5a. The two Bell states (Eq. (4)) are generated by two single-photon states, that are injected in two separate BSs. Then, Alice performs a partial BSM through a symmetric BS, analogously to the teleportation protocol described above. Bob and Charlie then retrieve their maximally entangled states $\left\vert {\psi }^{+}\right\rangle$ or $\left\vert {\psi }^{-}\right\rangle$ after Alice communicates the measurement outcome obtained at her stage, i.e., which of the two detectors counts. The success probability of the protocol is again *p*_±_ = 1/4. Certification of the output state after the entanglement swapping is then obtained via a further BSM between the modes at Bob’s and Charlie’s stages.

#### Experimental apparatus

To perform the photonic entanglement swapping protocol in Fig. 5a, we employ the setup shown in Fig. 5b. Unlike the teleportation protocol, we set the excitation laser power such to solely generate *π*-pulses and consider the initial state ${\left\vert 1\right\rangle }^{t = \tau }{\left\vert 1\right\rangle }^{t = 0}$. Then, a symmetric BS generates two Bell states of the form given in Eq. (4) and highlighted in green and pink respectively in Fig. 5a, b, to stress their correspondence with the Bell pairs of the circuital scheme in Fig. 4. The first Bell pair, arriving at time *t* = 0, is distributed along modes 1 and 2 yielding the following dual-rail state:

5 $${\left\vert {\psi }^{+}\right\rangle }^{t = 0}=\frac{{\left\vert 1\right\rangle }_{1}{\left\vert 0\right\rangle }_{2}+{\left\vert 0\right\rangle }_{1}{\left\vert 1\right\rangle }_{2}}{\sqrt{2}}$$

The second pair arrives at time *t* = *τ* and is distributed along modes 3 and 4 analogously. Subsystems 2 and 3 are sent to Alice’s MZI to perform a partial BSM. Subsystems 1 and 4, instead, are respectively sent to Bob and Charlie, who can be located, in principle, at an arbitrarily large distance. At this point, the spatial modes employed are four, as in Fig. 5a. The remainder of the protocol is thus equivalent to a probabilistic entanglement swapping. Indeed, Alice performs a partial BSM between subsystems 2 and 3 through a delay *t* = *τ* on mode 2 that synchronizes the two pulses and the interference on the red BS in Fig. 5b. According to Alice’s outcome, Bob and Charlie will end up with the following maximally entangled state both in the time bin and the path degrees of freedom:

$${\left\vert {\psi }^{\pm }\right\rangle }_{swap}=\frac{{\left\vert 1\right\rangle }_{1}^{t = 0}{\left\vert 0\right\rangle }_{4}^{t = \tau }\pm {\left\vert 0\right\rangle }_{1}^{t = 0}{\left\vert 1\right\rangle }_{4}^{t = \tau }}{\sqrt{2}}$$

The above state has the same form as the one in Eq. (5) up to a unitary transformation, and is characterized through interference on a BS and a delay *t* = *τ* applied to mode 1. Then, we measure the visibility of the fringes recorded in the single-photon counts of Bob and Charlie station conditioned to the Alice’s measurement outcomes. As for the teleportation protocol, we consider a two-fold coincidence window equal to 1.5 ns. The probabilistic routing of the states lowers the success probability of the protocol to *p*_±_ = 1/16.

#### Swapping results

In Fig. 5c, d, we show the single-count traces observed when the output modes at Bob’s and Charlie’s stages interfere in a BS. Indeed, similarly to the teleportation protocol, the interference fringes measured at the output can be employed as a means to verify that the entanglement swapping procedure was successful. Respectively, the measured visibilities for the four possible two-fold events are:

6 $$\left\{\begin{array}{l}{V}_{{A}_{1},C}=0.942\pm 0.002\quad \\ {V}_{{A}_{1},B}=0.862\pm 0.002\quad \\ {V}_{{A}_{2},C}=0.879\pm 0.002\quad \\ {V}_{{A}_{2},B}=0.903\pm 0.002\quad \end{array}\right.{V}_{{\rm{ave}}}=0.896\pm 0.001.$$

As demonstrated in Supplementary Note V, in the ideal case the visibility of the state shared between Charlie and Bob at the output of the BS is 1. However, taking into account the partial distinguishability of photons, the expected visibility amounts to *V*^theo^ = 0.902 and, therefore, it is in good agreement with our experimental results. Small deviations of the individual visibilities from this ideal value are mainly due to non-ideal reflectivities of the BSs and differences in the coupling and detection efficiencies. We estimated the experimental visibility through the same method used in the previous protocol and illustrated in the Methods. The corresponding fidelity of the teleported entangled state can be computed with the formula reported in refs. ^21,25^ and it amounts to ${F}_{{\rm{ave}}}=\frac{1+{V}_{{\rm{ave}}}}{2}=0.9480\pm 0.0005$. The high swapping fidelity with the Bell state $\left\vert {\psi }^{+}\right\rangle$ mirrors the quality of the entangled resource and of the quantum channel used for both the entanglement swapping and quantum teleportation protocols reported above in this work. Therefore, this result further supports the conclusion that our teleportation protocol employs genuine quantum resources.

## Discussions

In this work, we addressed a fundamental open problem, corresponding to the quantum teleportation of a general qubit encoded in the Fock basis. Indeed, due to technological constraints, such a problem was addressed until now by using linear optics, and, in particular, BSM operations. Vacuum-one photon states were indeed generated by letting a photon impinge on a BS and taking one of the output modes as the target mode. Such a procedure results in the quantum teleportation of one subsystem of an entangled state, while not permitting the quantum teleportation of genuine qubit states in such an encoding without the use of ancillary modes. To address this issue, we exploited the nonlinear properties of a semiconductor QD optically excited through RF. Indeed, it was recently demonstrated that such a procedure yields the production of coherent superposition states of vacuum and photon-number states. Within such a platform, we were able to quantum teleport six different pure genuine vacuum-one photon states without employing any ancillary mode and achieving results in good agreement with expectations. Moreover, within this setup, we were able to successfully perform entanglement swapping thus further extending the potentialities of our scheme to more complex scenarios.

We believe that our findings may represent an important step toward the development of large-scale quantum networks based on photon-number basis encoding, an approach that is widely investigated in quantum computation^52,53^ and communication tasks^36,54–57^. Further improvements of our protocol regard the implementation of full BSMs, which are still very challenging to realize in every photonic setup and encoding. We foresee as a future perspective using nonlinearities to realize such a kind of measurement, considering the recent advances in the field^24,58^. Our results may encourage new applications of QD-based single-photon sources for several quantum information tasks.

## Methods

### Characterization of the single-photon source

We use a Hanbury-Brown-Twiss setup to measure the values of the second-order auto-correlation function *g*_2_(0) of our single-photon source and a MZI to measure the Hong-Ou-Mandel (HOM) visibility *V*_HOM_, for each measurement station. For the quantum teleportation experiment, we only have two participants, Alice and Bob. For the entanglement swapping experiment, instead, we also have Charlie. However, for both experiments, we call more generally Bob’s HOM visibility, the one characterizing the second MZI.

Typical values throughout the whole experiment are (see also the Supplementary Note VI):

$$\begin{array}{l}{g}_{2}^{{\rm{Alice}}}(0)\,=\,0.0146\pm 0.0006,\\ \,\,{g}_{2}^{{\rm{Bob}}}(0)\,=\,0.0192\pm 0.0007,\\ \quad{V}_{{\rm{HOM}}}^{{\rm{Alice}}}\,=\,0.9055\pm 0.0015,\\ \quad{V}_{{\rm{HOM}}}^{{\rm{Bob}}}\,=\,0.8987\pm 0.0012.\end{array}$$

Additionally, we characterized the conditional purity of the state generated by our single-photon source by measuring the fringe visibility for different values of the excitation power, as also suggested in ref. ^35^. Indeed, it is necessary to consider the generation of a mixed state of the form:

$$\rho =\lambda {\rho }_{{\rm{pure}}}+(1-\lambda ){\rho }_{{\rm{mixed}}}$$

Here ${\rho }_{{\rm{pure}}}=\left\vert \psi \right\rangle \left\langle \psi \right\vert$ is a pure state, analogous to the one in Eq. (1) and encoded in the photon-number basis, while *ρ*_mixed_ = diag{∣*α*∣^2^, ∣*β*∣^2^} is a mixture of the vacuum and one-photon populations. As demonstrated in ref. ^35^, one can extract the value of *λ* by observing the dependence of the fringe visibility *V* from the vacuum population in a self-homodyne detection. For a more detailed discussion about the estimation of the purity *λ* conditioned on detection events, see Supplementary Note I. In our apparatus, *λ* is retrieved from the slope of the linear fit performed on the data in the inset of Fig. 3a. It amounts to *λ* ≈ 0.98.

### Characterization of the probe state

We characterize the probe state by using an independent procedure with respect to the protocol implementation. In detail, we observe the interference of two same probe states at the output of a standard MZI (see Fig. 2c) in Alice’s station. The *α*^2^ coefficient is then computed from the measured fringe visibility by inverting the following formula:

$$V={\lambda }^{2}\sqrt{{V}_{{\rm{HOM}}}^{{\rm{Alice}}}}{\alpha }^{2},$$

where *λ* is the state conditional purity. We report its full formal derivation in Supplementary Note I.

### Characterization of the teleported state and experimental imperfections

We characterize the teleported state by observing the fringe visibility at the output of Bob’s last BS. In the hypothesis of correct teleportation, which means that the state ${\left\vert \psi \right\rangle }_{{\rm{probe}}}$ and the teleported state have the same vacuum population *α*^2^, we expect that *V*_*T*_ = *V*. The imperfections of the experimental apparatus, such as the use of threshold detectors and of probabilistic routing of the states $\left\vert \psi \right\rangle$ and ${\left\vert \psi \right\rangle }_{{\rm{probe}}}$, limit the value of the measured *V*_*T*_, such that *V*_*T*_ < *V*. By taking into account such limitations and considering the other imperfections summarized by $\lambda ,{V}_{{\rm{HOM}}}^{{\rm{Alice}}}$ and ${V}_{{\rm{HOM}}}^{{\rm{Bob}}}$, we derive the following formula for the visibility of the teleported state as a function of *V*:

$${V}_{T}=\frac{2{\lambda }^{2}\sqrt{{V}_{{\rm{HOM}}}^{{\rm{Alice}}}{V}_{{\rm{HOM}}}^{{\rm{Bob}}}}V}{3{\lambda }^{2}\sqrt{{V}_{{\rm{HOM}}}^{{\rm{Alice}}}}-V}$$

The full derivation of such an equation is reported in Supplementary Note IV. The calculation has been carried out in the regime of high losses as for the derivation of *V*.

### Data analysis

We derived a procedure that improves significantly the accuracy of the measured visibility, even in the case of low event statistics, that we briefly discuss below. For details on the derivation we refer to Supplementary Note VII. We indicate with *n*(*ϕ*(*t*)) a single-photon count trace, where *ϕ*(*t*) is the freely evolving phase in a MZI. The visibility can be evaluated as:

$$V=\sqrt{2\frac{{\langle {n}^{2}\rangle }_{t}-{\langle n\rangle }_{t}^{2}-{\langle n\rangle }_{t}}{{\langle n\rangle }_{t}^{2}}}$$

where 〈⋅〉_*t*_ is the time average, and for brevity we have omitted in the notation the dependence of *n* on *ϕ*(*t*). The measured single-photon count traces for the teleportation (Fig. 3b) and entanglement swapping (Fig. 5) were recorded for a time of ~10^5^ bins of 50 ms that is around 1 h. The average number 〈*n*〉 of photons in each time bin ranges from 5 to 100 depending on the vacuum population.

## Supplementary information

Supplementary Information

## Data Availability

The data that support the findings of this study are available from the corresponding author upon reasonable request.
