# Supplementary material for: Teleportation of a genuine single-rail vacuum-one-photon qubit generated via a quantum dot source
Source: Npj Nanophoton. 2024 Nov 29;1(1):45. doi: 10.1038/s44310-024-00046-1 (PMC11606915; doi:10.1038/s44310-024-00046-1)
Supplement: Supplementary file 1 — Supplementary Information [file 44310_2024_46_MOESM1_ESM.pdf]

Supplementary Information: Teleportation of a genuine single-rail vacuum–one-photon qubit generated via a quantum dot source

Beatrice Polacchi,[1] Francesco Hoch,[1] Giovanni Rodari,[1] Stefano Savo,[1]
Gonzalo Carvacho,[1] Nicolò Spagnolo,[1] Taira Giordani,[1,*] and Fabio Sciarrino[1]
[1]*Dipartimento di Fisica - Sapienza Università di Roma, P.le Aldo Moro 5, I-00185 Roma, Italy*

I. CHARACTERIZATION OF THE PROBE STATE VIA SELF-HOMODYNING IN A MACH-ZEHNDER INTERFEROMETER

In this Section, we describe how the vacuum population of our target states is formally linked to their measured fringe visibility when measured in a time-unbalanced MZI. These results will allow us to extrapolate the purity of the states generated by our source, as well as to find the relationship between the target state visibility V and the visibility of the teleported state V_T, as reported in the next section.

We start by considering the quantum state entering the MZI, which is composed, in principle, by a train of n photon states. However, due to the losses in our setup, mainly caused by a low source extraction ($\approx 10\%$) and detection efficiency ($\approx 30\%$), we map the interferometer of Fig. 2c in the main text as if the two states $|\psi\rangle = \alpha|0\rangle + \beta|1\rangle$ and $|\psi\rangle_\phi = \alpha|0\rangle + \beta e^{i\phi}|1\rangle$, respectively, were impinging on a beam splitter, with high losses at the output. Under these assumptions, the input state is:

$$|\psi_{\text{in}}\rangle = |\psi\rangle|\psi\rangle_\phi = \left(\alpha + \beta a_1^\dagger\right)\left(\alpha + \beta e^{i\phi} a_2^\dagger\right)|0\rangle \tag{1}$$

After the beam splitter, the state evolves into:

$$|\psi_{\text{out}}\rangle = \left(\alpha + \beta\frac{a_1^\dagger + a_2^\dagger}{\sqrt{2}}\right)\left(\alpha + \beta e^{i\phi}\frac{a_1^\dagger - a_2^\dagger}{\sqrt{2}}\right)|0\rangle = \left[\alpha^2 + \frac{\alpha\beta(1+e^{i\phi})}{\sqrt{2}}a_1^\dagger + \frac{\alpha\beta(1-e^{i\phi})}{\sqrt{2}}a_2^\dagger + \frac{\beta^2 e^{i\phi}}{2}(a_1^{\dagger 2} - a_2^{\dagger 2})\right]|0\rangle\,. \tag{2}$$

Considering that, for threshold detectors, a single photon has probability η to be detected, while two-photon inputs trigger the detector with probability $1-(1-\eta)^2$, we find that the single-photon detection probability is:

$$P(\phi)_{|\psi\rangle} = \eta\frac{|\alpha|^2|\beta|^2}{2}(2+2\cos\phi) + \frac{|\beta|^4}{4}2[\eta^2 + 2\eta(1-\eta)] = \frac{\eta\beta^2}{2}(2 - |\beta|^2\eta + 2|\alpha|^2\cos\phi), \tag{3}$$

being η the overall loss parameter. By defining the visibility of the fringe pattern as:

$$V = \frac{\max_\phi P(\phi)_{|\psi\rangle} - \min_\phi P(\phi)_{|\psi\rangle}}{\max_\phi P(\phi)_{|\psi\rangle} + \min_\phi P(\phi)_{|\psi\rangle}}, \tag{4}$$

we find that, in the limit of high losses, ($\eta \to 0$) the visibility is:

$$V = |\alpha|^2. \tag{5}$$

This result can be extended to include partial photon distinguishability between the photon modes. This is obtained by considering an input state of the form:

$$|\psi_{\text{in}}^{x_1,x_2}\rangle = \left(\alpha + \beta\sqrt{x_A}a_1^\dagger + \beta\sqrt{1-x_A}b_1^\dagger\right)\left(\alpha + \beta e^{i\phi}\sqrt{x_B}a_2^\dagger + \beta e^{i\phi}\sqrt{1-x_B}c_2^\dagger\right)|0\rangle\,, \tag{6}$$

where $b_1^\dagger$ and $c_2^\dagger$ are two additional fictious modes, mutually orthogonal and orthogonal to $(a_1^\dagger, a_2^\dagger)$, that are used to model the effect of partially distinguishability due to the photon internal degrees of freedom. Parameters (x_A, x_B) are related to the Hong-Ou-Mandel visibility between the two photons as $V_{\text{HOM}} = x_A x_B$. Moreover, we include the purity of the state generated by the source.

* taira.giordani@uniroma1.it

Indeed, the unnormalized density matrix that our source generates, when considering the detection efficiency η, would read:

$$|0\rangle\langle 0| + \eta\left(\lambda\rho_{pure} + (1-\lambda)|1\rangle\langle 1|\right) + \mathcal{O}(\eta^2)$$

where all incoherent vacuum contributions (photon losses) are included in the first term and we neglect the $\mathcal{O}(\eta^2)$ terms, since $\eta \to 0$. Since, with our detection scheme, we have no way to measure the incoherent vacuum contributions, then the remaining state, conditioned to event detections, reads:

$$\rho = \lambda\rho_{\text{pure}} + (1-\lambda)\rho_{\text{mixed}} \quad (7)$$

where $\rho_{\text{pure}} = |\psi\rangle\langle\psi|$ and $\rho_{\text{mixed}} = |\alpha|^2|0\rangle\langle 0| + |\beta|^2|1\rangle\langle 1|$. In this sense, we use the term "conditional" purity. This parameter does not depend on the exact amount of losses [1]. By repeating the calculation above, and by tracing over the internal degrees of freedom of the photons, the fringe visibility of the state at the output of the interferometer is found to be $V = \lambda^2\sqrt{x_A x_B}|\alpha|^2$. We finally obtain:

$$V = \lambda^2\sqrt{V_{\text{HOM}}}|\alpha|^2 \quad (8)$$

This relationship can be employed to λ, from the linear fit reported in the inset of Fig. 3a of the main text. Indeed, by considering also the high loss regime, the measured signal rate is proportional to:

$$S_c \propto \langle P_1(\phi)\rangle_\phi \propto (1-\alpha^2) \quad (9)$$

which means that the visibility V is by itself linearly proportional to S_c, with slope dependent from the purity λ.

II. CHARACTERIZATION OF THE ENTANGLED STATES VIA A MACH-ZEHNDER INTERFEROMETER

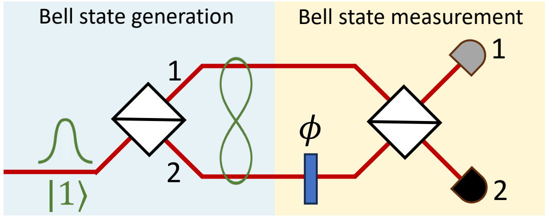

Figure 1. **Bell state generation and analyzer setup.** The first beam splitter generates the Bell state while the second one is used to perform a partial Bell state measurement.

The quantum resource required for a genuine teleportation is an entangled state shared by Alice and Bob. Here, we demonstrate that Alice and Bob share such quantum resources. Indeed, we measured the fidelity of our entangled state by using the setup shown in Fig. 1. which is a standard Mach-Zehnder interferometer. The Bell state $|\psi^+\rangle$ is generated by sending one photon in the first unbiased beam splitter while the Bell state measurement (BSM) is performed by the second one. More precisely, the ideal state after the first symmetric beam splitter is the following:

$$|\psi^+\rangle = \frac{|10\rangle_{12} + |01\rangle_{12}}{\sqrt{2}} \quad (10)$$

The second beam splitter mirrors implements the inverse transformation of the first one such that the detection probability of one photon at output modes 1,2 oscillates as $P_{1,\text{click}} = \cos^2(\phi/2)$; $P_{2,\text{click}} = \sin^2(\phi/2)$. The oscillating phase factor ϕ is only due to the optical path difference in the Mach-Zehnder interferometer. Consequently, the fidelity of our experimental entangled state with the Bell state $|\psi^+\rangle$ is given by the $\max_\phi P^{exp}_{1,\text{click}}(\phi)$. At the experimental level, the value of the fidelity with the Bell state can be retrieved from the measurement of the visibility V of the fringes at the output of the MZI, according to the relationship $F = \frac{1+V}{2}$. Such a derivation and argument is largely discussed in Refs. [2, 3], the two works that reported the first implementations of a vacuum-one-photon teleportation protocol. Discrepancies from the ideal fidelity are mainly due to a partially asymmetric beam splitter and a non-vanishing $g^2(0)$ value. In our setup the MZI fringes visibility is near unity, thus demonstrating, on one hand, the presence of coherence in the superposition state in (10), and on the other, the high fidelity with the Bell state. The high fidelity obtained ensures that Alice and Bob share an entangled resource and, therefore, that the teleportation protocol here reported exploits genuine quantum resources and would not be reproducible with only classical resources.

III. CLASSICAL BOUND FOR THE FRINGE VISIBILITY

We now discuss the formal derivation of the visibility one would observe when using the classical strategy reported in [4] and known as the *measure-and-prepare* protocol. From [4], we know that the state closest to the target that Alice can prepare without performing a full quantum state tomography of the qubit is the following:

$$\rho_{CT} = F\left|\psi\right\rangle\!\left\langle\psi\right| + (1-F)\left|\psi_\perp\right\rangle\!\left\langle\psi_\perp\right| \tag{11}$$

where the parameter F is the fidelity with the target state $|\psi\rangle$, which can only take values in $[1/3, 2/3]$, while $|\psi_\perp\rangle = \beta^*|0\rangle - \alpha^*|1\rangle$. In particular, in the best case, this qubit will have $F = 2/3$ with the target state $|\psi\rangle$ and can also be written as $\rho_{CT} = \frac{1}{3}|\psi\rangle\langle\psi| + \frac{1}{3}|0\rangle\langle 0| + \frac{1}{3}|1\rangle\langle 1|$. Consequently, in such a case, the fidelity with $|\psi_\perp\rangle$ is $F = 1/3$.

Considering our implementation, the classical bound can be retrieved by evaluating the fringe visibility recorded in Bob's station when a probe qubit $|\psi\rangle_\phi$ and the state ρ_{CT} interfere. The single-photon detection probability in this case reads:

$$P(\phi)_{\rho_{CT}} = FP(\phi)_{|\psi\rangle} + (1-F)P(\phi)_{|\psi_\perp\rangle} \tag{12}$$

The term $P(\phi)_{|\psi\rangle}$ was derived in Supplementary Note I and is defined in Eq. (3). Instead, the detection probability when the states $|\psi\rangle_\phi$ and $|\psi_\perp\rangle$ interfere reads:

$$P(\phi)_{|\psi_\perp\rangle} = \frac{\eta}{2}(1 - 2|\alpha|^2|\beta|^2\cos\phi) - \frac{|\alpha|^2|\beta|^2}{2}\eta^2 \tag{13}$$

In the high-loss limit ($\eta \longrightarrow 0$), the fringe visibility reads:

$$V_{\rho_{CT}} = \frac{2|\alpha|^2|\beta|^2|2F-1|}{1+F(2|\beta|^2-1)} \tag{14}$$

which, using $V = |\alpha|^2$ (derived in Eq. (5) of Supplementary Note I), becomes:

$$V_{\rho_{CT}} = \frac{2V(1-V)|2F-1|}{1+F(1-2V)} \tag{15}$$

We analyze $V_{\rho_{CT}}$ as a function of V in Supplementary Fig. 2, for different values of the fidelity F. We first observe that, for fixed values of F, the curve is always monotonously decreasing for $V \in [0, 1/2]$ and monotonously increasing for $V \in [1/2, 1]$. In particular, for $F = 1/2$, the curve $V_{\rho_{CT}}(V)$ is constantly zero. We also notice that, for $F \in (1/3, 2/3)$, all curves are bounded by zero and the red and the blue plots. As an example, we show the curves for five different values of the fidelity $F = \{1/2,\ 3/5,\ 2/5,\ 3/7,\ 3/8\} \in (1/3, 2/3)$. We highlighted this area in purple to represent the convolution of the two curves for $F = 1/3$ and $F = 2/3$. This means that the whole classically accessible region (highlighted in purple) is delimited by the value zero and the function $\max_F\{V_T(V,F)\}$, which define the bound achievable by using the classical strategy *measure-and-prepare*. This region is also highlighted in purple in Fig. 3a of the main text.

IV. FULL MODEL OF THE QUANTUM TELEPORTATION SCHEME

In this section, we develop a model to describe the expected visibility of states teleported with our platform, V_T, and discuss its relationship with the visibility of the target state V. We will refer to the spatial modes, to the beam-splitters (BS), and to the delay loops as labeled in Fig. 3.

We first consider an initial state where partial photon indistinguishability is not included, as:

$$|\psi\rangle_{3,2\tau} \otimes |1\rangle_{1,\tau} \otimes |\psi\rangle_{3,0} = (\alpha|0\rangle + \beta|1\rangle)_{3,2\tau} \otimes |1\rangle_{1,\tau} \otimes (\alpha|0\rangle + \gamma|1\rangle)_{3,0} = \left(\alpha + \beta a^\dagger_{3,2\tau}\right) a^\dagger_{1,\tau} \left(\alpha + \gamma a^\dagger_{3,0}\right)|0\rangle\,, \tag{16}$$

The qubit we want to teleport is $|\psi\rangle = \alpha|0\rangle + \gamma|1\rangle$, where $\gamma = |\beta|e^{i\chi}$, $\beta = |\beta|e^{i\phi}$ and $\phi - \chi = \delta$.

After application of BS_{12} the state evolves into:

$$\left(\alpha + \beta a^\dagger_{3,2\tau}\right)\frac{1}{\sqrt{2}}\left(a^\dagger_{1,\tau} + a^\dagger_{2,\tau}\right)\left(\alpha + \gamma a^\dagger_{3,0}\right)|0\rangle\,. \tag{17}$$

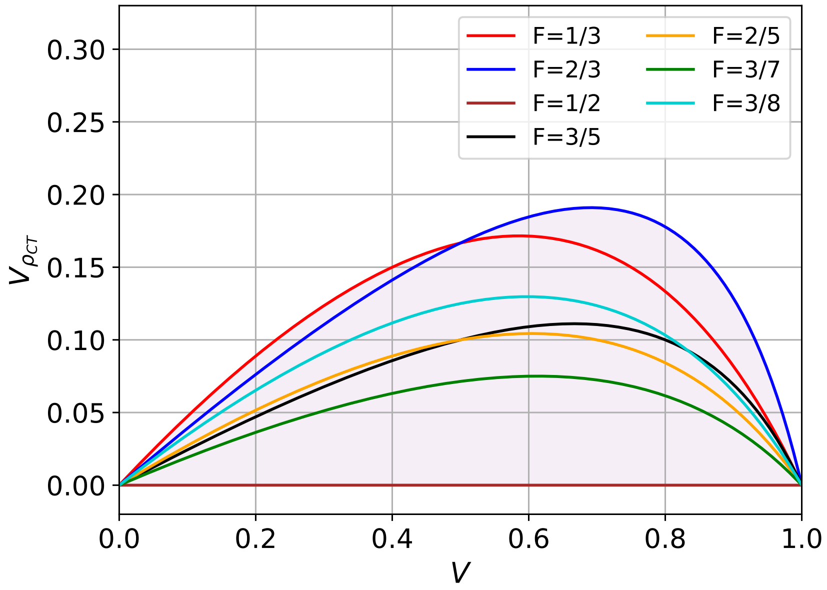

Figure 2. **Classical bound on the fringe visibility in the *measure-and-prepare* protocol. a)** The purple area defines the classical region for the visibility of the state ρ_{CT} ($V_{\rho_{CT}}$) in terms of the visibility of the target state (V), using the *measure-and-prepare* strategy. This region is bounded by the red and blue curves, corresponding respectively to $F = 1/3$ and $F = 2/3$. For $F \in (1/3, 2/3)$, all curves are below the red and blue ones, which therefore represents the classical bound on $V_{\rho_{CT}}$.

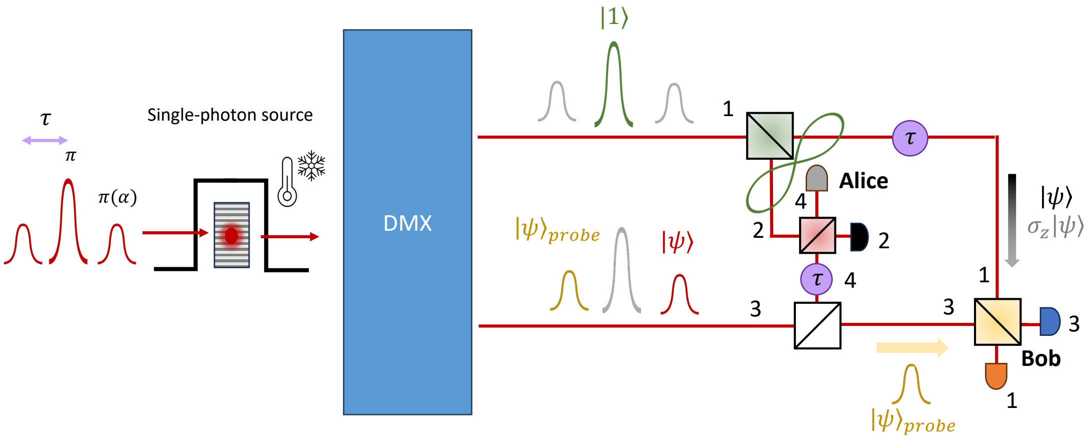

Figure 3. **Teleportation scheme.** Here we recall the teleportation scheme, already reported in the main text, by adding the modes labels used for the calculations carried out in this section.

This operation transforms the qubit $|1\rangle_{1,\tau}$ in the following Bell state over modes 1 and 2:

$$|1\rangle_{1,\tau} \rightarrow \frac{\left(|1\rangle^{\mathrm{B}}_{1,\tau}\,|0\rangle^{\mathrm{A}}_{2,\tau} + |0\rangle^{\mathrm{B}}_{1,\tau}\,|1\rangle^{\mathrm{A}}_{2,\tau}\right)}{\sqrt{2}} \tag{18}$$

where we highlighted with the superscripts A and B that mode 1 travels to Bob while mode 2 travels to Alice. Then, after application of BS_{34} we find:

$$\left(\alpha + \beta\frac{a^\dagger_{3,2\tau} + a^\dagger_{4,2\tau}}{\sqrt{2}}\right)\frac{1}{\sqrt{2}}\left(a^\dagger_{1,\tau} + a^\dagger_{2,\tau}\right)\left(\alpha + \gamma\frac{a^\dagger_{3,0} + a^\dagger_{4,0}}{\sqrt{2}}\right)|0\rangle\,. \tag{19}$$

After application of delay τ on mode 4 we obtain:

$$\left(\alpha+\beta\frac{a^{\dagger}_{3,2\tau}+a^{\dagger}_{4,3\tau}}{\sqrt{2}}\right)\frac{1}{\sqrt{2}}\left(a^{\dagger}_{1,\tau}+a^{\dagger}_{2,\tau}\right)\left(\alpha+\gamma\frac{a^{\dagger}_{3,0}+a^{\dagger}_{4,\tau}}{\sqrt{2}}\right)|0\rangle \tag{20}$$

At this point, Alice performs a Bell state measurement between the qubit to be teleported, i.e. $|\psi\rangle$, and the subsystem of the Bell pair that she kept. Indeed, after application of BS_{24}, we find:

$$\left(\alpha+\frac{\beta}{2}(\sqrt{2}a^{\dagger}_{3,2\tau}+a^{\dagger}_{2,3\tau}-a^{\dagger}_{4,3\tau})\right)\frac{1}{2}\left(\sqrt{2}a^{\dagger}_{1,\tau}+a^{\dagger}_{2,\tau}+a^{\dagger}_{4,\tau}\right)\left(\alpha+\frac{\gamma}{2}(\sqrt{2}a^{\dagger}_{3,0}+a^{\dagger}_{2,\tau}-a^{\dagger}_{4,\tau})\right)|0\rangle \tag{21}$$

Let us now analyze the state in which the qubit outgoing from mode 1 of BS_{12} and traveling to Bob is left after Alice's Bell state measurement. According to the teleportation protocol, we expect this qubit to be in a state identical to $|\psi\rangle$, up to a unitary transformation. We can temporarily neglect the term in the first bracket and consider only the evolution of subsystems $|\psi\rangle_{3,0}$ and $|1\rangle_{1,\tau}$:

$$\frac{1}{2}\left(\sqrt{2}a^{\dagger}_{1,\tau}+a^{\dagger}_{2,\tau}+a^{\dagger}_{4,\tau}\right)\left(\alpha+\frac{\gamma}{2}(\sqrt{2}a^{\dagger}_{3,0}+a^{\dagger}_{2,\tau}-a^{\dagger}_{4,\tau})\right)|0\rangle \tag{22}$$

When post-selecting on the occurrence of one detection event in Alice's detector 2 at time τ, we are left with the following state:

$$\frac{1}{2}\left(\alpha a^{\dagger}_{2,\tau}+\frac{\gamma}{\sqrt{2}}a^{\dagger}_{2,\tau}a^{\dagger}_{1,\tau}+\frac{\gamma}{\sqrt{2}}a^{\dagger}_{3,0}a^{\dagger}_{2,\tau}+\frac{\gamma}{2}(a^{\dagger}_{2,\tau})^2\right)|0\rangle \tag{23}$$

The term $\frac{\gamma}{\sqrt{2}}a^{\dagger}_{3,0}a^{\dagger}_{2,\tau}$ appears due to the fact that we are employing non-deterministic photon routing to divide $|\psi\rangle$ and $|\psi\rangle_{probe}$. Moreover, the two-photon contribution $\frac{\gamma}{2}(a^{\dagger}_{2,\tau})^2$ cannot be distinguished experimentally from a single detection event without using photon-number-resolving detectors. If we could experimentally exclude such terms (with active photon routing and photon-number-resolving detectors), the teleported state appearing on mode 1 would read:

$$\rho_T^{\text{ideal}}=\begin{pmatrix}|\alpha|^2 & \alpha\gamma^*\\ \alpha^*\gamma & |\gamma|^2\end{pmatrix} \tag{24}$$

which is identical to the state $\rho=|\psi\rangle\langle\psi|$ that we aim to teleport. In this ideal situation, the fringe visibility V_T at the output of Bob's self-homodyne measurement would be identical to visibility V of the state to be teleported, estimated via a standard MZI-based technique. We show this ideal case in the grey plot in Fig. 3a of the main text.

However, when taking into account those two terms, the normalized state traveling along mode 1 reads:

$$\rho_T=\frac{1}{|\alpha|^2+\frac{3}{2}|\gamma|^2}\begin{pmatrix}|\alpha|^2+|\gamma|^2 & \frac{\alpha\gamma^*}{\sqrt{2}}\\ \frac{\alpha^*\gamma}{\sqrt{2}} & \frac{|\gamma|^2}{2}\end{pmatrix} \tag{25}$$

Analogous reasoning can be applied to the case in which one detection event occurs in Alice's detector 4 at time τ, where the final state would read:

$$\rho_T=\frac{1}{|\alpha|^2+\frac{3}{2}|\gamma|^2}\begin{pmatrix}|\alpha|^2+|\gamma|^2 & -\frac{\alpha\gamma^*}{\sqrt{2}}\\ -\frac{\alpha^*\gamma}{\sqrt{2}} & \frac{|\gamma|^2}{2}\end{pmatrix} \tag{26}$$

The difference between the state in Eq. (24) and the ones in Eq. (25)-(26) causes the observed fringe visibility at Bob's station to be different from the ideal case $V_T=V$.

We now continue with the calculations to quantify the expected visibility V_T in such a non-ideal case, which is shown in the green plot of Fig. 3a of the main text. After application of delay τ on mode 1 we obtain:

$$\left(\alpha+\frac{\beta}{2}(\sqrt{2}a^{\dagger}_{3,2\tau}+a^{\dagger}_{2,3\tau}-a^{\dagger}_{4,3\tau})\right)\frac{1}{2}\left(\sqrt{2}a^{\dagger}_{1,2\tau}+a^{\dagger}_{2,\tau}+a^{\dagger}_{4,\tau}\right)\left(\alpha+\frac{\gamma}{2}(\sqrt{2}a^{\dagger}_{3,0}+a^{\dagger}_{2,\tau}-a^{\dagger}_{4,\tau})\right)|0\rangle \tag{27}$$

Finally, after application of BS_{13} the state can be written as:

$$\left[\alpha+\frac{\beta}{2}(a^\dagger_{3,2\tau}+a^\dagger_{1,2\tau}+a^\dagger_{2,3\tau}-a^\dagger_{4,3\tau})\right]\frac{1}{2}\left(a^\dagger_{3,2\tau}-a^\dagger_{1,2\tau}+a^\dagger_{2,\tau}+a^\dagger_{4,\tau}\right)\left[\alpha+\frac{\gamma}{2}(a^\dagger_{3,0}+a^\dagger_{1,0}+a^\dagger_{2,\tau}-a^\dagger_{4,\tau})\right]|0\rangle \tag{28}$$

The output state at the detection can be also rearranged as:

$$\begin{aligned}
&\frac{\alpha^2}{2}\left[a^\dagger_{3,2\tau}-a^\dagger_{1,2\tau}+a^\dagger_{2,\tau}-a^\dagger_{4,\tau}\right]+\frac{\alpha\gamma}{4}\left[\left(a^\dagger_{3,2\tau}-a^\dagger_{1,2\tau}\right)\left(a^\dagger_{3,0}+a^\dagger_{1,0}\right)+\left(a^\dagger_{3,2\tau}-a^\dagger_{1,2\tau}\right)\left(a^\dagger_{2,\tau}-a^\dagger_{4,\tau}\right)+\right.\\
&\left.+\left(a^\dagger_{2,\tau}+a^\dagger_{4,\tau}\right)\left(a^\dagger_{3,0}+a^\dagger_{1,0}\right)+\left(a^\dagger_{2,\tau}+a^\dagger_{4,\tau}\right)\left(a^\dagger_{2,\tau}-a^\dagger_{4,\tau}\right)\right]+\frac{\alpha\beta}{4}\left[\left(a^\dagger_{3,2\tau}+a^\dagger_{1,2\tau}\right)\left(a^\dagger_{3,2\tau}-a^\dagger_{1,2\tau}\right)+\right.\\
&\left.+\left(a^\dagger_{2,3\tau}-a^\dagger_{4,3\tau}\right)\left(a^\dagger_{2,\tau}+a^\dagger_{4,\tau}\right)+\left(a^\dagger_{3,2\tau}+a^\dagger_{1,2\tau}\right)\left(a^\dagger_{2,\tau}+a^\dagger_{4,\tau}\right)+\left(a^\dagger_{2,3\tau}-a^\dagger_{4,3\tau}\right)\left(a^\dagger_{3,2\tau}-a^\dagger_{1,2\tau}\right)\right]+\\
&+\frac{\beta\gamma}{8}\left[\left(a^\dagger_{3,2\tau}+a^\dagger_{1,2\tau}\right)\left(a^\dagger_{3,2\tau}-a^\dagger_{1,2\tau}\right)\left(a^\dagger_{3,0}+a^\dagger_{1,0}\right)+\left(a^\dagger_{3,2\tau}+a^\dagger_{1,2\tau}\right)\left(a^\dagger_{3,2\tau}-a^\dagger_{1,2\tau}\right)\left(a^\dagger_{2,\tau}-a^\dagger_{4,\tau}\right)+\right.\\
&+\left(a^\dagger_{3,2\tau}+a^\dagger_{1,2\tau}\right)\left(a^\dagger_{2,\tau}+a^\dagger_{4,\tau}\right)\left(a^\dagger_{3,0}+a^\dagger_{1,0}\right)+\left(a^\dagger_{3,2\tau}+a^\dagger_{1,2\tau}\right)\left(a^\dagger_{2,\tau}+a^\dagger_{4,\tau}\right)\left(a^\dagger_{2,\tau}-a^\dagger_{4,\tau}\right)+\\
&+\left(a^\dagger_{2,3\tau}-a^\dagger_{4,3\tau}\right)\left(a^\dagger_{3,2\tau}-a^\dagger_{1,2\tau}\right)\left(a^\dagger_{3,0}+a^\dagger_{1,0}\right)+\left(a^\dagger_{2,3\tau}-a^\dagger_{4,3\tau}\right)\left(a^\dagger_{3,2\tau}-a^\dagger_{1,2\tau}\right)\left(a^\dagger_{2,\tau}-a^\dagger_{4,\tau}\right)+\\
&\left.+\left(a^\dagger_{2,3\tau}-a^\dagger_{4,3\tau}\right)\left(a^\dagger_{2,\tau}+a^\dagger_{4,\tau}\right)\left(a^\dagger_{3,0}+a^\dagger_{1,0}\right)+\left(a^\dagger_{2,3\tau}-a^\dagger_{4,3\tau}\right)\left(a^\dagger_{2,\tau}+a^\dagger_{4,\tau}\right)\left(a^\dagger_{2,\tau}-a^\dagger_{4,\tau}\right)\right]|0\rangle
\end{aligned} \tag{29}$$

From this expression, we can know the probability of having an event where one detector per station clicks. More specifically, we need to consider coincidences event between one detector of the pair (1,3), clicking at time 2τ, and one detector of the pair (2,4) clicking at time τ. The four combinations for all probabilities are found to be:

$$\begin{aligned}
P(1,2)&=\frac{|\beta|^2}{16}\left(2-2|\alpha|^2\cos\delta\right),\\
P(1,4)&=\frac{|\beta|^2}{16}\left(2+2|\alpha|^2\cos\delta\right),\\
P(3,2)&=\frac{|\beta|^2}{16}\left(2+2|\alpha|^2\cos\delta\right),\\
P(3,4)&=\frac{|\beta|^2}{16}\left(2-2|\alpha|^2\cos\delta\right),
\end{aligned} \tag{30}$$

leading to the following visibility:

$$V_T=|\alpha|^2 \tag{31}$$

Until this point, we did not take into account other experimental effects such as losses, purity, and partial indistinguishability. However, analogously to the previous section, we need to consider also such different effects. We begin by including the effect of losses in our setup, analogously to the derivation above. Since the loss operator commutes with the other operators, we can compute the teleported visibility in a lossy platform, by including all the losses in the detector efficiency. With respect to the previous derivation, the probabilities of the interesting events become:

$$\begin{aligned}
P(1_{2\tau},2_\tau)&=\frac{|\beta|^2\eta_1\eta_2}{32}\left(6-\eta_1-\eta_2-(2-\eta_1-\eta_2)|\alpha|^2-4|\alpha|^2\cos\delta\right),\\
P(1_{2\tau},4_\tau)&=\frac{|\beta|^2\eta_1\eta_4}{32}\left(6-\eta_1-\eta_4-(2-\eta_1-\eta_4)|\alpha|^2+4|\alpha|^2\cos\delta\right),\\
P(3_{2\tau},2_\tau)&=\frac{|\beta|^2\eta_3\eta_2}{32}\left(6-\eta_3-\eta_2-(2-\eta_3-\eta_2)|\alpha|^2+4|\alpha|^2\cos\delta\right),\\
P(3_{2\tau},4_\tau)&=\frac{|\beta|^2\eta_3\eta_4}{32}\left(6-\eta_3-\eta_4-(2-\eta_3-\eta_4)|\alpha|^2-4|\alpha|^2\cos\delta\right),
\end{aligned} \tag{32}$$

where $\eta_1\ldots\eta_4$ are the detection probabilities at the four outputs. In the limit $\eta_i\to 0$, all probabilities have the same visibility which is found to be:

$$V_T=\frac{2|\alpha|^2}{3-|\alpha|^2} \tag{33}$$

Finally, with analogous procedure to the one discussed in the previous section, we now take into account also the state non-ideal purity $\lambda < 1$ and HOM visibilities. Hence, the formula in Eq. (33) becomes:

$$V_T = \frac{2\lambda^2 \sqrt{V_{\text{HOM}}^{\text{Alice}} V_{\text{HOM}}^{\text{Bob}}} |\alpha|^2}{3 - |\alpha|^2}. \tag{34}$$

In this way, by using Eq. (5), the relationship between the target state visibility V and the visibility V_T of the teleported state in our platform can be written as:

$$V_T = \frac{2\lambda^2 \sqrt{V_{\text{HOM}}^{\text{Alice}} V_{\text{HOM}}^{\text{Bob}}} V}{3\lambda^2 \sqrt{V_{\text{HOM}}^{\text{Alice}}} - V}. \tag{35}$$

This expression has been used as the green line in Fig. 3a of the main text to identify the expected visibility from the collected experimental data. The experimental values of the target state visibilities and vacuum populations, and teleported visibilities, are reported in Table I.

$\lvert\alpha\rvert^2$	V	V_T
0.211 ± 0.001	0.197 ± 0.001	0.13 ± 0.02
0.323 ± 0.005	0.303 ± 0.005	0.21 ± 0.02
0.425 ± 0.002	0.398 ± 0.002	0.26 ± 0.02
0.545 ± 0.006	0.510 ± 0.006	0.36 ± 0.03
0.632 ± 0.004	0.591 ± 0.004	0.41 ± 0.04
0.769 ± 0.005	0.720 ± 0.005	0.52 ± 0.05

Table I. Comparison between the visibility of the teleported state V_T with the visibility of the target state V with vacuum population $|\alpha|^2$, estimated independently via the self-homodyning procedure in a MZI.

V. FULL MODEL OF THE ENTANGLEMENT SWAPPING EXPERIMENT

In this section, we derive the model employed to describe the expected visibility of the output state after the entanglement swapping protocol. In this derivation, we first consider the case where the two interfering states are two single-photon states, and the BS transmittance is $T = 0.5$. Then, we will consider a noise model where the states at the input are allowed to have a nonzero vacuum component. We refer to Fig. 4 for mode labelling and for the sequence of operations considered in the model.

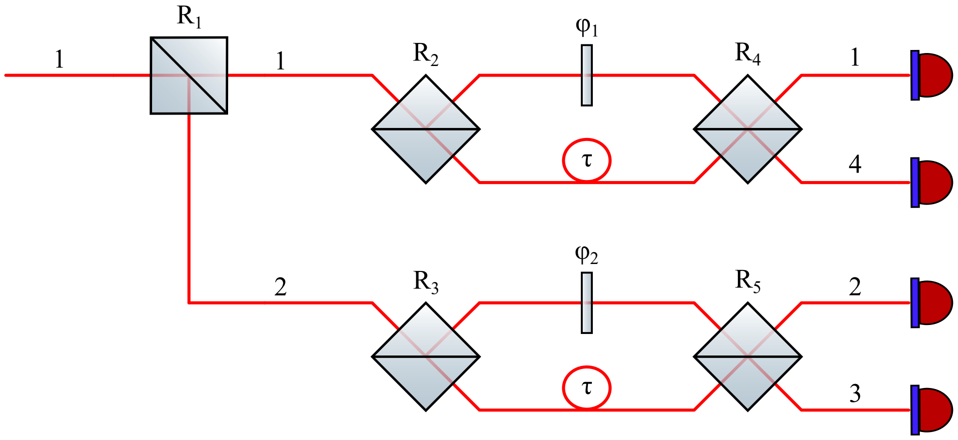

Figure 4. **Entanglement swapping apparatus.** Scheme of the optical modes and beam-splitters reflectivies ($R_i = 1 - T_i$)to perform the entanglement swapping.

A. Single-photon input case

We consider here the ideal case where all BS in Fig. 4 have transmittance $t_i^2 = T_i = 0.5$, and the input state is a set of two single photons:

$$|1\rangle_\tau |1\rangle_0 . \tag{36}$$

Such a state can be written through creation operators as:

$$\left[a^\dagger_{1,\tau}\right]\left[a^\dagger_{1,0}\right]|0\rangle . \tag{37}$$

After propagation through the first BS, that acts as $a_1^\dagger \to \left(a_1^\dagger + a_2^\dagger\right)/\sqrt{2}$, the state becomes:

$$\frac{1}{2}\left[a^\dagger_{1,\tau} + a^\dagger_{2,\tau}\right]\left[a^\dagger_{1,0} + a^\dagger_{2,0}\right]|0\rangle . \tag{38}$$

Then, after the first layer of two BS evolutions, the state will evolve into:

$$\frac{1}{4}\left[a^\dagger_{1,\tau} + a^\dagger_{4,\tau} + a^\dagger_{3,\tau} + a^\dagger_{2,\tau}\right]\left[a^\dagger_{1,0} + a^\dagger_{4,0} + a^\dagger_{3,0} + a^\dagger_{2,0}\right]|0\rangle . \tag{39}$$

Before the last layer of two BSs, we observe that:

- mode 1 and 2 will acquire a time delay equal to τ;
- mode 4 and mode 3 will acquire a relative phase shift $e^{i\phi}$ and $e^{i\phi'}$ respectively, due to the propagation in the Mach-Zehnder interferometer.

The state before the final layer of BSs becomes:

$$\frac{1}{4}\left[a^\dagger_{1,2\tau} + e^{i\phi}a^\dagger_{4,\tau} + e^{i\phi'}a^\dagger_{3,\tau} + a^\dagger_{2,2\tau}\right]\left[a^\dagger_{1,\tau} + e^{i\phi}a^\dagger_{4,0} + e^{i\phi'}a^\dagger_{3,0} + a^\dagger_{2,\tau}\right]|0\rangle . \tag{40}$$

By looking at a specific time bin $t = \tau$, we can neglect the other terms and keep only the following part of the state:

$$\frac{1}{4}\left[e^{i\phi}a^\dagger_{4,\tau} + e^{i\phi'}a^\dagger_{3,\tau}\right]\left[a^\dagger_{1,\tau} + a^\dagger_{2,\tau}\right]|0\rangle . \tag{41}$$

By applying the evolution of the final layer of two BSs, we obtain that the relevant part of the final state reads:

$$\frac{1}{8}\left[e^{i\phi}a^\dagger_{4,\tau} + e^{i\phi}a^\dagger_{1,\tau} + e^{i\phi'}a^\dagger_{2,\tau} - e^{i\phi'}a^\dagger_{3,\tau}\right]\left[a^\dagger_{4,\tau} - a^\dagger_{1,\tau} + a^\dagger_{2,\tau} + a^\dagger_{3,\tau}\right]|0\rangle . \tag{42}$$

The probabilities $\mathrm{P}_{4,2}$, $\mathrm{P}_{4,3}$, $\mathrm{P}_{1,2}$, $\mathrm{P}_{1,3}$ for the relevant two-fold coincidence events $\mathrm{CC}_{4,2}$, $\mathrm{CC}_{4,3}$, $\mathrm{CC}_{1,2}$, $\mathrm{CC}_{1,3}$, can be then obtained as:

$$P^{\mathrm{ID}}_{1,2} = \frac{1}{32}\left(1 - cos\xi\right); \qquad P^{\mathrm{ID}}_{1,3} = \frac{1}{32}\left(1 + cos\xi\right); \qquad P^{\mathrm{ID}}_{4,2} = \frac{1}{32}\left(1 + cos\xi\right); \qquad P^{\mathrm{ID}}_{4,3} = \frac{1}{32}\left(1 - cos\xi\right). \tag{43}$$

where $\xi = \phi - \phi'$. In this ideal scenario, all visibilities are then equal to 1.

B. Model of the experiment

We now consider the scenario where the two states contain a small superposition with a vacuum component. The input state is then written as:

$$\left[\alpha|0\rangle + \beta|1\rangle\right]_\tau \left[\alpha|0\rangle + \gamma|1\rangle\right]_0 , \tag{44}$$

where γ is related to β through a phase shift $\gamma = e^{i\delta}\beta$. Expressing the one-photon state through the creation operators, the initial state is written as:

$$\left[\alpha + \beta a^\dagger_{1,\tau}\right]\left[\alpha + \gamma a^\dagger_{1,0}\right]|0\rangle . \tag{45}$$

We then follow the same assumptions of the ideal case, except that the transmittance of the BSs can be different than 0.5. Therefore, the first BS would transform the creation operators as $a_1^\dagger \to \left(t_1 a_1^\dagger + r_1 a_2^\dagger\right)$, and the state becomes:

$$\left[\alpha + \beta\left(t_1 a_{1,\tau}^\dagger + r_1 a_{2,\tau}^\dagger\right)\right]\left[\alpha + \gamma\left(t_1 a_{1,0}^\dagger + r_1 a_{2,0}^\dagger\right)\right]|0\rangle\,. \tag{46}$$

Then, by considering the evolution induced by BS_2 and BS_3, we obtain the following state:

$$\left[\alpha + \beta t_1\left(t_2 a_{1,\tau}^\dagger + r_2 a_{4,\tau}^\dagger\right) + \beta r_1\left(r_3 a_{3,\tau}^\dagger + t_3 a_{2,\tau}^\dagger\right)\right]\left[\alpha + \gamma t_1\left(t_2 a_{1,0}^\dagger + r_2 a_{4,0}^\dagger\right) + \gamma r_1\left(r_3 a_{3,0}^\dagger + t_3 a_{2,0}^\dagger\right)\right]|0\rangle\,, \tag{47}$$

that can be also expanded:

$$\left[\alpha + \beta t_1 t_2 a_{1,\tau}^\dagger + \beta t_1 r_2 a_{4,\tau}^\dagger + \beta r_1 r_3 a_{3,\tau}^\dagger + \beta r_1 t_3 a_{2,\tau}^\dagger\right]\left[\alpha + \gamma t_1 t_2 a_{1,0}^\dagger + \gamma t_1 r_2 a_{4,0}^\dagger + \gamma r_1 r_3 a_{3,0}^\dagger + \gamma r_1 t_3 a_{2,0}^\dagger\right]|0\rangle\,. \tag{48}$$

As for the previous calculations, we have that:

- mode 1 and 2 will acquire a time delay equal to τ;
- mode 4 and mode 3 will acquire a relative phase shift $e^{i\phi}$ and $e^{i\phi'}$ respectively, due to the propagation in the Mach-Zehnder interferometer.

Hence, the state evolves into:

$$\begin{aligned}&\left[\alpha + \beta t_1 t_2 a_{1,2\tau}^\dagger + e^{i\phi}\beta t_1 r_2 a_{4,\tau}^\dagger + e^{i\phi'}\beta r_1 r_3 a_{3,\tau}^\dagger + \beta r_1 t_3 a_{2,2\tau}^\dagger\right]\times\\ &\times\left[\alpha + \gamma t_1 t_2 a_{1,\tau}^\dagger + e^{i\phi}\gamma t_1 r_2 a_{4,0}^\dagger + e^{i\phi'}\gamma r_1 r_3 a_{3,0}^\dagger + \gamma r_1 t_3 a_{2,\tau}^\dagger\right]|0\rangle\,.\end{aligned} \tag{49}$$

By keeping only terms related to time bin $t = \tau$, and by neglecting the vacuum components that will not lead to a useful two-fold coincidence events, we can write the relevant portion of the state as:

$$\left[e^{i\phi}\beta t_1 r_2 a_{4,\tau}^\dagger + e^{i\phi'}\beta r_1 r_3 a_{3,\tau}^\dagger\right]\left[\gamma t_1 t_2 a_{1,\tau}^\dagger + \gamma r_1 t_3 a_{2,\tau}^\dagger\right]|0\rangle\,. \tag{50}$$

After the evolution of the final layer of two BSs, the revelant part of the final state reads:

$$\begin{aligned}&\left[e^{i\phi}\beta t_1 r_2 r_4 a_{1,\tau}^\dagger + e^{i\phi}\beta t_1 r_2 t_4 a_{4,\tau}^\dagger + e^{i\phi'}\beta r_1 r_3 r_5 a_{2,\tau}^\dagger - e^{i\phi'}\beta r_1 r_3 t_5 a_{3,\tau}^\dagger\right]\times\\ &\times\left[\gamma t_1 t_2 r_4 a_{4,\tau}^\dagger - \gamma t_1 t_2 t_4 a_{1,\tau}^\dagger + \gamma r_1 t_3 t_5 a_{2,\tau}^\dagger + \gamma r_1 t_3 r_5 a_{3,\tau}^\dagger\right]|0\rangle\,.\end{aligned} \tag{51}$$

We can now obtain the probabilities $\mathrm{P}_{4,2}$, $\mathrm{P}_{4,3}$, $\mathrm{P}_{1,2}$, $\mathrm{P}_{1,3}$ for the relevant two-fold coincidence events $\mathrm{CC}_{4,2}$, $\mathrm{CC}_{4,3}$, $\mathrm{CC}_{1,2}$, $\mathrm{CC}_{1,3}$.

- For Channels 1 and 2 we obtain:

$$P_{1,2} = |\beta|^2 R_1 T_1\left(R_2 T_3 R_4 T_5 + T_2 R_3 T_4 R_5 + 2\sqrt{R_2 T_2 R_3 T_3 R_4 T_4 R_5 T_5}\cos\xi\right), \tag{52}$$

$$V_{1,2} = \frac{2\sqrt{R_2 T_2 R_3 T_3 R_4 T_4 R_5 T_5}}{R_2 T_3 R_4 T_5 + T_2 R_3 T_4 R_5}. \tag{53}$$

- For Channels 1 and 3 we obtain:

$$P_{1,3} = |\beta|^2 R_1 T_1\left(R_2 T_3 R_4 R_5 + T_2 R_3 T_4 T_5 + 2\sqrt{R_2 T_2 R_3 T_3 R_4 T_4 R_5 T_5}\cos\xi\right), \tag{54}$$

$$V_{1,3} = \frac{2\sqrt{R_2 T_2 R_3 T_3 R_4 T_4 R_5 T_5}}{R_2 T_3 R_4 R_5 + T_2 R_3 T_4 T_5}. \tag{55}$$

- For Channels 4 and 2 we obtain:

$$P_{4,2} = |\beta|^2 R_1 T_1\left(R_2 T_3 T_4 T_5 + T_2 R_3 R_4 R_5 + 2\sqrt{R_2 T_2 R_3 T_3 R_4 T_4 R_5 T_5}\cos\xi\right), \tag{56}$$

$$V_{4,2} = \frac{2\sqrt{R_2 T_2 R_3 T_3 R_4 T_4 R_5 T_5}}{R_2 T_3 T_4 T_5 + T_2 R_3 R_4 R_5}. \tag{57}$$

- For Channels 4 and 3 we obtain

$$P_{4,3} = |\beta|^2 R_1 T_1 \left(R_2 T_3 T_4 R_5 + T_2 R_3 R_4 T_5 + 2\sqrt{R_2 T_2 R_3 T_3 R_4 T_4 R_5 T_5} \cos\xi \right), \tag{58}$$

$$V_{4,3} = \frac{2\sqrt{R_2 T_2 R_3 T_3 R_4 T_4 R_5 T_5}}{R_2 T_3 T_4 R_5 + T_2 R_3 R_4 T_5}. \tag{59}$$

As an additional source of noise, we also take into account partially distinguishable photons with HOM visibility V_{HOM}. The result is that all four visibility are scaled of the same factor $m = V_{HOM}$.

Let us now define the following four parameters:

$$x := \frac{T_2 R_3}{R_2 T_3}; \qquad y := \frac{T_4}{R_4}; \qquad z := \frac{T_5}{R_5}; \qquad w := 2m\sqrt{xyz}. \tag{60}$$

In this way, we can rewrite the four visibilities as:

$$\begin{cases} V_{1,2} = \frac{w}{xy+z}, \\ V_{1,3} = \frac{w}{xyz+1}, \\ V_{4,2} = \frac{w}{x+yz}, \\ V_{4,3} = \frac{w}{y+xz}. \end{cases} \tag{61}$$

Through this parametrization, we are able to retrieve some relations between the visibilities, the reflectivity of the BSs and the photons indistinguishability parameters. Indeed, after some calculations, we retrieve:

$$\begin{cases} z = \frac{t_2+t_3-t_1^2-1 \pm \sqrt{(t_2+t_3-t_1^2-1)^2 - 4(t_2 t_3 - t_1)^2}}{2(t_2 t_3 - t_1)}, \\ x = \frac{t_2 - t_3 z}{t_1 - z}, \\ y = \frac{t_3 - z t_2}{t_1 - z}, \\ w = \frac{V_{1,2}(1-z^2)}{t_1 - z}, \end{cases} \qquad \begin{cases} t_1 := \frac{V_{1,2}}{V_{1,3}}, \\ t_2 := \frac{V_{1,2}}{V_{4,2}}, \\ t_3 := \frac{V_{1,2}}{V_{4,3}}. \end{cases} \tag{62}$$

where the sign is chosen such that $z \geq 0$. We will use such relations to validate our experimental results, as described in the following subsection.

C. Entanglement swapping results analysis

We first report the measured visibilities in our apparatus:

$$\begin{cases} V_{A_1,C} = 0.942 \pm 0.002 \\ V_{A_1,B} = 0.862 \pm 0.002 \\ V_{A_2,C} = 0.879 \pm 0.002 \\ V_{A_2,B} = 0.903 \pm 0.002 \end{cases} \qquad V_{\text{ave}} = 0.896 \pm 0.001. \tag{63}$$

obtained at the four output combinations of the entanglement swapping protocol. To validate the experiment, we compare these results with the model derived above. We first assume that the BS reflectivities have the ideal values:

$$R_2 = R_3 = R_4 = R_5 = 0.5, \tag{64}$$

while $V_{HOM} = 0.902$ is the Hong-Ou-Mandel visibility averaged over the two MZI interferometers. In this case, we find that the expected visibility for all possible two-fold events amounts to $V^{\text{theo}} = 0.902$. The difference between the theoretical prediction and the experimental one can be attributed to unbalanced splitting ratios of the various BSs and to different coupling and detection efficiencies in the apparatus. All these effects can be summarized by considering non-ideal reflectivities of the BSs.

If we follow the inverse approach, using the equations 62 and deriving the reflectivities of the BSs from the experimental visibilities we obtain:

$$x = 1.16 \pm 0.11; \qquad R_4 = 0.44 \pm 0.04; \qquad R_5 = 0.38 \pm 0.08; \qquad V_{HOM} = 0.92 \pm 0.04. \tag{65}$$

These value are compatible with the parameters of the experimental apparatus.

VI. CHARACTERIZATION OF THE SINGLE-PHOTON SOURCE

We characterize the single-photon purity of our source by measuring the second-order auto-correlation function in a Hanbury-Brown-Twiss interferometer at both Alice's and Bob's stations. Here, we show the normalized coincidences as a function of the delay in one of the two arms of the interferometer for both stations in Fig. 5a-b. The single-photon indistinguishability is characterized by measuring the HOM visibility of two consecutive photons in a MZI interferometer as the one shown in Fig. 2b of the main text, for both Alice's and Bob's stations. Here, we show the normalized coincidences as a function of the delay in one of the two arms of the interferometer for both stations in Fig. 5c-d.

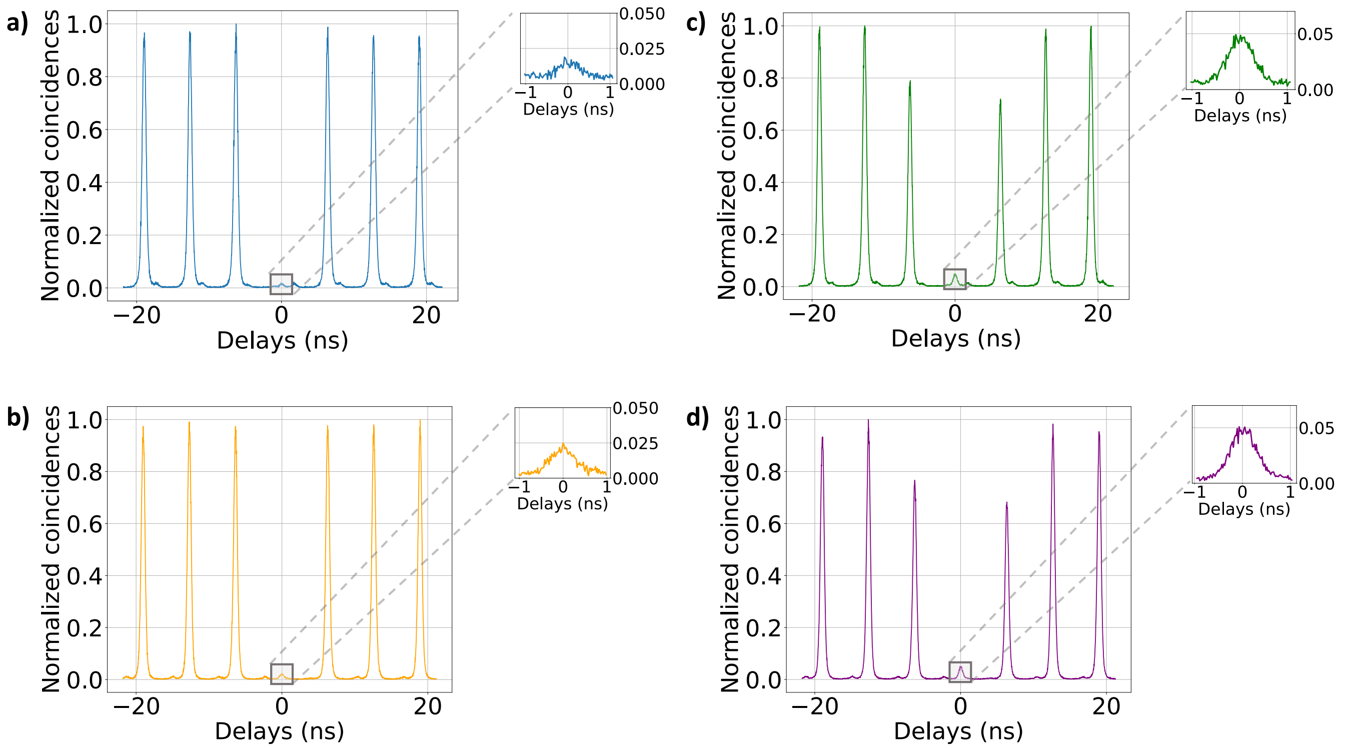

Figure 5. **Characteristics of our source. a)-b)** Plot of the normalized coincidences against time delay in a Hanbury-Brown-Twiss interferometer for the measurement of the second-order autocorrelation $g_2(0)$ of respectively Alice's and Bob's stations. **c)-d)** Plot of the normalized coincidences against time delay between in a Mach-Zehnder interferometer for the measurement of the HOM visibility V_{HOM} of respectively Alice's and Bob's stations.

Finally, we report typical values of such quantities throughout the whole experiment:

$$\begin{aligned} g_2^{\text{Alice}}(0) &= 0.0146 \pm 0.0006 \\ g_2^{\text{Bob}}(0) &= 0.0192 \pm 0.0007 \\ V_{\text{HOM}}^{\text{Alice}} &= 0.9055 \pm 0.0015 \\ V_{\text{HOM}}^{\text{Bob}} &= 0.8987 \pm 0.0012 \end{aligned} \tag{66}$$

VII. ESTIMATION OF THE VISIBILITY FROM THE SINGLE-PHOTON COUNTS

In this section, we report the derivation of a reliable procedure that we employed to estimate the fringe visibility. As discussed here, such a method allows an accurate estimation of the visibility of a single-count trace even in the presence of low statistics, whereas standard methods would significantly overestimate it, as depicted in Fig. 6. A single-count time trace can be expressed as:

$$n(t) = \mathcal{P}\left(N\frac{1+V\cos(\phi(t))}{2}\right) \tag{67}$$

where $\mathcal{P}(\cdot)$ is a sample from a Poissonian distribution with mean value equal to the quantity in the parenthesis, $\phi(t)$ is a stochastic variable that varies slowly compared to signal sampling times, N is the average number of photons and V is the fringes visibility of the signal that is the parameter to be estimated.

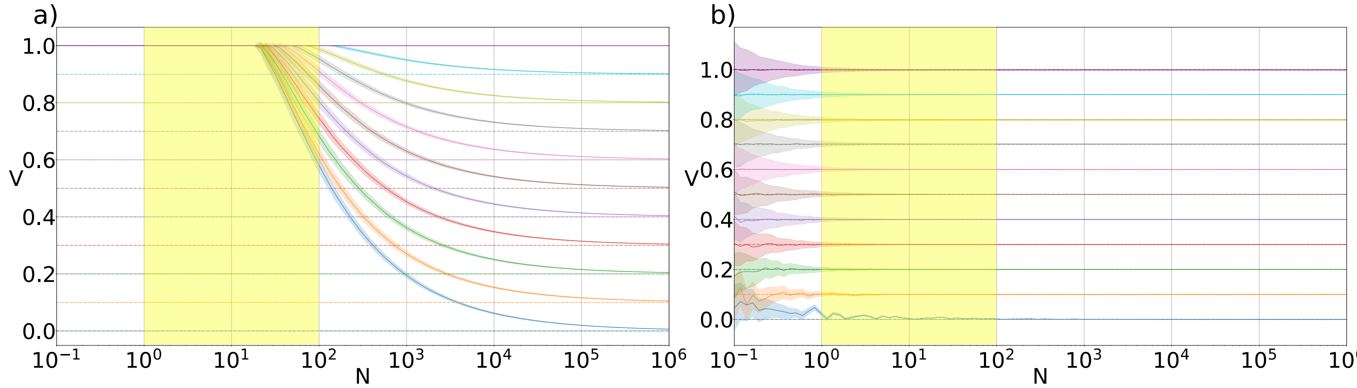

Figure 6. **Estimation of fringes visibility.** Numerical simulation of the visibility measurement for a typical time trace of the experiment that covers 10^5 time bins of 50ms, as a function of the average number of photons N in each time bin. The dashed line represents the true visibility of the fringes. The solid line is the mean value reconstructed by averaging on 1000 instances of the simulation. The shaded regions represent the variance of such estimations. In a) the visibility is estimated according to Eq. (68); b) represents the reconstruction based on the measure of the trace variance. The region highlighted in yellow is the range of the N in our teleportation experiment.

The standard procedure to measure the visibility of a single-count trace, as the one in Eq. (67), consists of applying the following formula:

$$V = \frac{\max(n(t)) - \min(n(t))}{\max(n(t)) + \min(n(t))} \tag{68}$$

but, as shown in Fig. 6a, if the number of photons N is small, then a significant bias is obtained in the estimation of the visibility, especially in the region of the teleportation and entanglement swapping experiments (that is, $N = 10^0 \div 10^2$, highlighted in yellow in the figure).

To overcome this issue, we employed a different approach based on the measurement of the signal variance. Under the reasonable assumption that $\langle\cos(\phi(t))\rangle_t = 0$ and $\langle\cos(2\phi(t))\rangle_t = 0$, and that the time trace is sufficiently long and hence statistically significant, then we can compute the time average of the signal and of the square of the signal as:

$$\langle n\rangle_t = \left\langle N\frac{1+V\cos(\phi(t))}{2}\right\rangle = \frac{N}{2} \tag{69}$$

$$\langle n^2\rangle_t = \left\langle \left(N\frac{1+V\cos(\phi(t))}{2}\right)^2\right\rangle + \left\langle N\frac{1+V\cos(\phi(t))}{2}\right\rangle = \frac{N^2}{4}\left(1+\frac{V^2}{2}\right) + \frac{N}{2} \tag{70}$$

By combining previous equations we obtain:

$$V^2 = 2\frac{\langle n^2\rangle_t - \langle n\rangle_t^2 - \langle n\rangle_t}{\langle n\rangle_t^2} \tag{71}$$

As we can observe, in Fig. 6b this approach is more reliable compared to the previous one, due to the fast convergence of the estimated value of the visibility in the regime of the low values of N, i.e. of low statistics.

[1] J. Loredo, C. Antón, B. Reznychenko, P. Hilaire, A. Harouri, C. Millet, H. Ollivier, N. Somaschi, L. De Santis, A. Lemaître, I. Sagnes, L. Lanco, A. Auffèves, O. Krebs, and P. Senellart, "Generation of non-classical light in a photon-number superposition," Nature Photonics, vol. 13, no. 11, pp. 803–808, 2019.
[2] E. Lombardi, F. Sciarrino, S. Popescu, and F. De Martini, "Teleportation of a vacuum–one-photon qubit," Phys. Rev. Lett., vol. 88, no. 7, p. 070402, 2002.

[3] D. Fattal, E. Diamanti, K. Inoue, and Y. Yamamoto, “Quantum teleportation with a quantum dot single photon source,” Phys. Rev. Lett., vol. 92, no. 3, p. 037904, 2004.
[4] S. Massar and S. Popescu, “Optimal extraction of information from finite quantum ensembles,” Phys. Rev. Lett., vol. 74, pp. 1259–1263, Feb 1995.
